# Optimization of 1,4-Naphthoquinone Hit Compound: A Computational, Phenotypic, and In Vivo Screening against *Trypanosoma cruzi*

**DOI:** 10.3390/molecules26020423

**Published:** 2021-01-15

**Authors:** Leonardo S. Lara, Guilherme C. Lechuga, Caroline dos S. Moreira, Thaís B. Santos, Vitor F. Ferreira, David R. da Rocha, Mirian C. S. Pereira

**Affiliations:** 1 Laboratório de Ultraestrutura Celular, Instituto Oswaldo Cruz, Fiocruz, Av. Brasil 4365 Manguinhos, Rio de Janeiro 21040-900, RJ, Brazil; leonardosilva.lara@hotmail.com (L.S.L.); guilherme.lechuga@yahoo.com.br (G.C.L.); 2 Departamento de Química Orgânica, Instituto de Química, Universidade Federal Fluminense, Rua Outeiro São João Batista, Niterói 24020-141, Rio de Janeiro, Brazil; carolmoreira13@gmail.com (C.d.S.M.); Barreto.thais2006@gmail.com (T.B.S.); vitorferreira@id.uff.br (V.F.F.); davidrocha@id.uff.br (D.R.d.R.)

**Keywords:** *Trypanosoma cruzi*, naphthoquinones, trypanocidal activity, chemotherapy, compound optimization

## Abstract

Chagas disease (CD) still represents a serious public health problem in Latin America, even after more than 100 years of its discovery. Clinical treatments (nifurtimox and benznidazole) are considered inadequate, especially because of undesirable side effects and low efficacy in the chronic stages of the disease, highlighting the urgency for discovering new effective and safe drugs. A small library of compounds (**1a**–**i** and **2a**–**j**) was designed based on the structural optimization of a Hit compound derived from 1,4-naphthoquinones (C2) previously identified. The biological activity, structure-activity relationship (SAR), and the in silico physicochemical profiles of the naphthoquinone derivatives were analyzed. Most modifications resulted in increased trypanocidal activity but some substitutions also increased toxicity. The data reinforce the importance of the chlorine atom in the thiophenol benzene ring for trypanocidal activity, highlighting **1g,** which exhibit a drug-likeness profile, as a promising compound against *Trypanosoma cruzi*. SAR analysis also revealed **1g** as cliff generator in the structure-activity similarity map (SAS maps). However, compounds C2 and **1g** were unable to reduce parasite load, and did not prevent mouse mortality in *T. cruzi* acute infection. Phenotypic screening and computational analysis have provided relevant information to advance the optimization and design of new 1,4-naphthoquinone derivatives with a better pharmacological profile.

## 1. Introduction

Chagas disease (CD), a serious parasitic disease caused by *T. cruzi*, continues to be a neglected disease with a high social and economic impact in Latin America, despite the control strategies of the World Health Organization’s programs launched to combat this disease [1]. Unlike other neglected tropical diseases (NTDs), little progress has been achieved in the treatment and diagnosis of CD, with the pediatric formulation of benznidazole (Bz) [2], the reference drug, and the recent approval of Bz by the United States Food and Drug Administration [3] being the major advances to date.

Despite improved control of vector-borne transmission in wide areas of Southern Cone countries, outbreaks of oral infection and the appearance of secondary peridomestic vectors have resulted in the re-emergence of acute infection in many endemic countries [4,5,6]. Non-endemic countries have also faced this silent disease because of the emigration of asymptomatic infected individuals, who are unaware that they harbor *T. cruzi*, making disease control a major challenge for the local public policies and hence, a global public health problem [7].

The limitations of the drugs used in the clinical treatment, Bz and nifurtimox, call attention to the elements possibly involved with the therapeutic failure, including (i) Serious adverse effects, leading to discontinuation of treatment [8]; (ii) the genetic variability of *T. cruzi* and the presence of naturally resistant strains [9,10]; (iii) the state of dormancy of *T. cruzi* and its resistance to treatment [11] and the low efficacy of Bz in chronic phase [12]. In this scenario, the search for new therapeutic regimens of reference drugs, aiming to preserve efficacy and minimize the side effects, as well as the identification of new targets, potent and safe drugs are urgently needed for the treatment of CD. Clinical trials have been critical in deepening the knowledge about the efficacy of Bz in the chronic phase and its synergistic action in combined therapy with promising new drugs, as well as in evaluating the efficacy of new or repositioned drugs as control strategies to fight DC. The BENEFIT (Benznidazole Evaluation for Interrupting Trypanosomiasis) trial revealed the progress of chronic chagasic cardiomyopathy even after treatment with Bz [13]; the lack of efficacy of Posaconazole monotherapy and combined therapy with Bz has been demonstrated in the Chagazol [14] and Stop-Chagas [15] clinical trials, respectively. The phase II proof of concept of fexinidazole showed a reduction of the parasitic burden at low doses but the treatment was discontinued because of safety and tolerability [16]. Recently, the benznidazole New Doses Improved Treatment and Associations (BENDITA) trial, which demonstrated low-dose efficacy of Bz in a short-course regimen, offered new hope for treatment with good efficacy and reduced side effects [17], but prevention of cardiomyopathy progression is yet to be evaluated.

Efforts in the design and discovery of new drugs led to the identification of several hit and lead compounds that advanced for optimization in an attempt to achieve more effective trypanocidal drugs [18]. Virtual computational predictions have guided promising candidates to move forward to preclinical in vivo assays [19]. In silico analyses have highlighted multiple physicochemical parameters as a strategy to improve molecules with greater potential to successfully overcome adverse toxicological outcomes [20] and even alert to possible risks that may contribute to compound failure [21]. The analysis of the structure-activity relationship (SAR) has also been exploited in the design of new drugs, providing valuable information about how structural changes can improve potency and bioavailability [22].

Naphthoquinone derivatives, including 2-phenoxy-1,4-naphthoquinone, have been proposed as an attractive anti-trypanosomal phenotypic hit attributable to their multitarget mode of action [23,24]. Naphthoquinones, an important class of natural products of wide biological application, have been reported to inhibit glyceraldehyde-3-phosphate dehydrogenase (GAPDH) intervening in the glycolytic pathway [25], inhibiting tubulin polymerization and exhibiting antiproliferative activity [26], and inducing oxidative stress by generating reactive oxygen species (ROS) [27,28]. In this subject area, we have previously reported the 2-hydroxy-3-phenylsulfanylmethyl-[1,4]-naphthoquinone derivative, compound 2 (C2), as a potential hit compound [29]. C2 showed no cardiotoxic effect and trypanocidal activity against clinically relevant forms of *T. cruzi*. Thus, a small library of 1,4-naphthoquinone derivatives (19 derivatives) was designed aiming to explore the structural activity relations (SAR) of C2 and optimize its trypanocidal activity. Based on the structure of C2, in which we noted the importance of the chlorine atom in the thiophenol benzene ring, we employed the previously reported anti-*T. cruzi* activity of *para*- chlorobenzene group [29] and analyzed the effect generated by both the introduction of more chlorine atoms, and varying their position in the final structure. Knowing that one of the main mechanisms of action of naphthoquinones is related to the generation of ROS [28] and considering our previous findings that demonstrated greater ROS generation by the introduction of a hydroxyl group in the aromatic ring of the naphthoquinones [29], we also evaluated the effect caused by this pattern in C2. Furthermore, the introduction of 4-CH_3_ and 4-OCH_3_-phenylgroups considered C2′s ability to potentiate the trypanocidal activity, as described in lapachone-derived naphthoimidazoles and naphthofuranquinones compared to parental compounds [30,31]. The presence of methylthio group (SCH_3_) has been associated with the redox potential of several naphthoquinones, since these compounds can participate in oxidation reactions forming reactive sulfur species [32]. Other substituents, including fluoro (F), nitro (-NO_2_), propyl (C_3_H_7_), and naphthyl (C_10_H_8_) groups were added in order to improve the therapeutic properties.

In this study, a small library of 1,4-naphthoquinone derivatives was synthesized and screened against *T. cruzi*. Also, a computational multi-parameter analysis was performed to predict physicochemical properties, analyze structure-activity relationship and systematically characterize the activity landscape of 1,4-naphthoquinone derivatives against *T. cruzi*, allowing us to determine structural changes associated with activity differences, in order to identify a promising candidate for treatment of Chagas disease.

## 2. Results

### 2.1. Hit Compound Optimization

The series of 2-hydroxy-3-phenylsulfanylmethyl-[1,4]-naphthoquinone derivatives (**1a**–**f, 1h** and **1i**) was designed considering the results reported in previous studies of our research group, where we observed that the introduction of chlorine atoms in R1, combined with benzene in R2, promoted good anti-*T. cruzi* activity [29]. Inspired by these results, we started this study trying to improve the anti-*T. cruzi* activity by exploring the introduction of electron-withdrawing groups like chlorine in R1 (compounds **1a**–**d**) and/or electron-releasing groups like methoxy (OCH_3_; **1g**–**i**) in R2. Later, in order to better correlate anti-*T. cruzi* activity and the electronic profile, we designed a new series of compounds (**2a**–**j**) to study the effect of different substituents in R1 (CH_3_, NO_2_, F, OCH_3_, SCH_3_, C_3_H_7_, C_10_H_8_) in the presence of methoxy group, an electron-donating group, in the benzene ring (R2) [33]. Finally, we decided to observe if the introduction of a triazole in R2 (1-(3,5-dichlorophenyl)-1H-1,2,3-triazole, **1e**), a heterocycle extensively present in antiproliferative compounds [27], or presence of hydroxyl group in the quinonic aromatic ring (R3; **1f**), usually associated with increased ROS formation [34], could improve anti-*T. cruzi* activity.

### 2.2. In Vitro Biological Evaluation

The small library (**1a**–**i** and **2a**–**j**) was analyzed for its toxicity profile in mammalian cells and trypanocidal activity against trypomastigotes and intracellular amastigotes of two different *T. cruzi* lineages (TcI and TcII). The toxicity levels of the 1,4-naphthoquinone derivatives in Vero cells resulted in CC_50_ values ranging from 24.4 to 141.8 µM while Bz reached CC_50_ > 500 μM (Table 1). The compound **1h**, consisting of OCH_3_ in 2 (*ortho*) and 4 (*para*) positions of the benzene ring (R2), was the most cytotoxic of the series (CC_50_ = 24.4 ± 2.6 μM). Most compounds showed high toxicity, with a CC_50_ in the range of 34–49 μM, except compounds **1d** (82.1 ± 5.1 μM), **1g** (99.9 ± 12.5 μM), **2d** (141.8 ± 17.6 μM), and **2j** (93.5 ± 6.6 μM) (Table 1).

Regarding trypanocidal activity, both series were analyzed for effect on Dm28c expressing luciferase (Dm28c-Luc; TcI) and Y strain (TcII) trypomastigotes. Among the 19 derivatives screened, only eight compounds, six of series 1 (**1a**–**e** and **1g**) and two of series 2 (**2f** and **2g**), showed higher activity than Bz (Y strain − CC_50_ = 14.3 ± 3.2 μM; Dm28c-Luc − CC_50_ = 17.5 ± 3.3 μM) for both Y strain and Dm28c-Luc trypomastigotes (Table 1). Although compound **1e** was shown to be more effective (IC_50_ approximately 4.5 μM) against *T. cruzi* trypomastigotes, it presented a low selectivity index (SI) because of its high cytotoxicity to mammalian cells (CC_50_ = 34.1 ± 2.9 μM). Only compound **1g** (CC_50_ = 99.9 ± 12.5 μM) presented SI ≥ 9.8 for both *T. cruzi* stocks, reaching IC_50_ values of 1.4- to 2-fold lower than the Bz for Y strain (IC_50_ = 10.2 ± 0.5 μM) and Dm28c-Luc (IC_50_ = 8.1 ± 0.6 μM), respectively (Table 1). The IC_90_ reached values ≤ 19.3 μM for trypomastigotes (Y strain and Dm28c clone), showing greater efficacy than Bz (IC_90_ > 100 μM). The luminescent screening assay for naphthoquinone derivatives (**1a**–**i** and 2**a**–**j**) targeting intracellular amastigotes (Dm28c-Luc) revealed six most active derivatives (**1a**, **1b**, **1g**, **2a**, **2e**, and **2f**) (Table 2). Although these derivatives have IC_50_ values ≤6.9 µM, they showed lower activity than Bz (IC_50_ = 1.4 ± 0.4 µM). However, **1g** (IC_50_ = 6.7 ± 1.8 μM) was the only derivative with SI > 10 among the most active compounds (Table 2).

### 2.3. Computational Analysis: Physicochemical Properties, SAR, and SAS Maps

**Figure 1:**
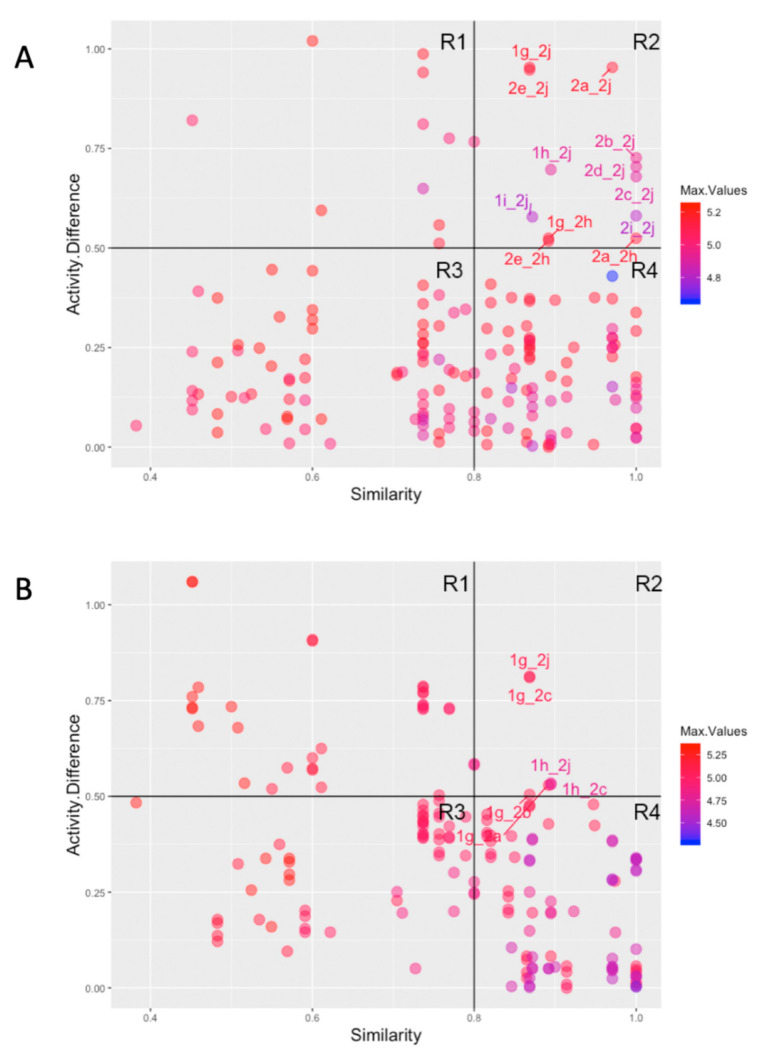
Structure-activity similarity maps (SAS maps) for *T. cruzi* based on chemical structure and biological activity. SAS maps against trypomastigote (**A**) and intracellular amastigote (**B**) forms of *T. cruzi*. Each point represents a pairwise comparison of 1,4-naphthoquinones derivatives. The points are colored to pIC50 values using a continuous scale from low (blue) to high (red) power. R1 = not descriptive; R2 = similar structure and different activity (Cliff activity); R3 = Different structure and similar activity (Similarity cliff) and R4 = Similar structure and activity (Smooth SAR).

The high structure similarity and high activity difference between molecules of the series led us to analyze structure-activity relationship (SAR), using the concept of activity landscape, and the physicochemical properties of all derivatives. In this light, structure-activity similarity (SAS) maps were analyzed with emphasis on the activity cliff that clusters structurally similar compound pairs with large activity differences. The SAS maps for the 19 derivatives against trypomastigotes and intracellular amastigotes revealed 172 data points with each one corresponding to a pairwise comparison (Figure 1). Data points were additionally differentiated by the potency (pIC50) using a color scale from low (blue) to high (red). Most of the compound pairs generated for trypomastigotes and intracellular amastigotes were identified in similarity cliff (region III; R3) and smooth SAR (region IV; R4), showing small structural similarity with high activity similarity and high structural and activity similarity, respectively (Figure 1). The quantitative analysis revealed that 86.5% and 77.7% of compound pairs were distributed in the R3 and R4 regions of SAS maps for both amastigotes and trypomastigotes, respectively (Figure 1). In contrast, the compound pairs’ lowest proportion of data points were in the activity cliff (region II; R2). A higher proportion of activity cliff pairs was identified for intracellular amastigotes (7%) compared to trypomastigotes (3.5%) (Figure 1). The pairs **1g**_**2j**, **1g**_**2c**, **1g**_**2a**, **1g**_**2b**, **1h**_**2j,** and **1h**_**2c** illustrate the activity cliffs in the SAS map of trypomastigotes. Data points **1g**_**2h**, **1g**_**2j**, **1h**_**2j**, **1i**_**2j**, **2a**_**2h, 2a**_**2j**, **2b**_**2j**, **2c**_**2j**, **2d**_**2j**, **2e**_**2j**, **2e**_**2h,** and **2i**_**2j** represent the activity cliffs of intracellular amastigotes (Figure 1). Interesting, two compound pairs, **1g**_**2j** and **1h**_**2j**, were identified in the activity cliffs in the SAS map of both trypomastigotes and intracellular amastigotes. Although small structural changes were evident among these molecules, **1g** is among the most active compounds while **2j** is the least effective compound in the series.

**Figure 2:**
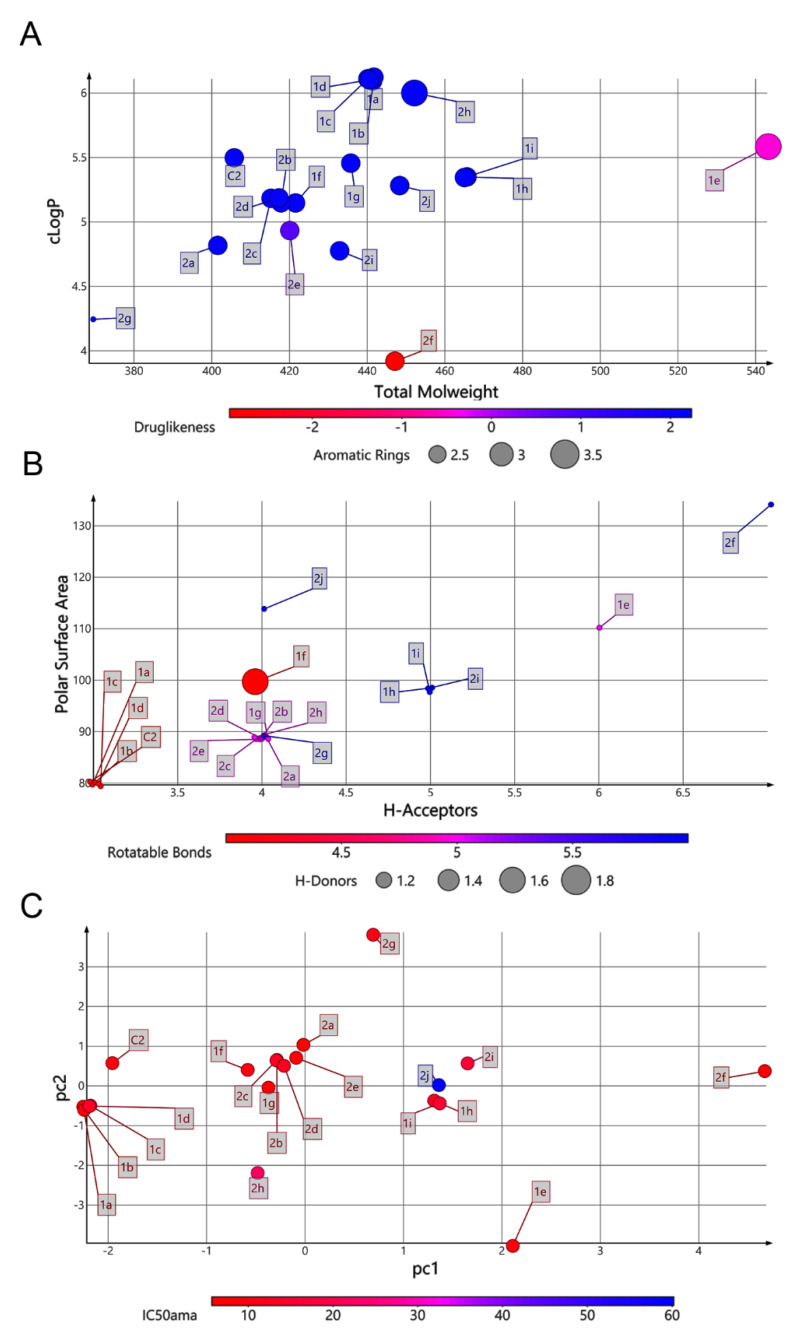
Physicochemical properties of 1,4-naphthoquinone series. Relationship of physicochemical properties between cLogP x Molecular Weight x Drug-likeness × number of aromatic rings (**A**); polar surface area x hydrogen bond acceptors x hydrogen bond donors x rotatable bonds (**B**,**C**) principal component analysis (PCA).

Thus, we addressed the question of how the structural changes in the series affected the physicochemical properties of the derivatives and resulted in drastic changes in compound activity. Then, we assessed the molecular properties typically analyzed in drug discovery software, including molecular mass (MM), lipophilicity (LogP), number of hydrogen donors (HBDs) and acceptors (HBAs), polar surface area (PSA), rotatable bonds, pIC50 and drug-likeness, using Datawarrior software. These properties are important to determine compound absorption, distribution, metabolism, and excretion (ADME) profile. The series **1(a**–**i)** has the highest MW (418.46–542.82) compared to the series **2(a**–**j)**, which achieved maximal of 452.42 (Figure 2). Most of the compounds have high lipophilicity (LogP > 4.27), except for **2f** with moderate lipophilicity (LogP = 3.9), but they do not violate Lipinski’s rule of five (Ro5) (Figure 2). Hydrogen bonds, which increase aqueous solubility, also fit the Ro5 with HBD < 5 and HBA < 10. The PSA values, associated with permeability and oral absorption prediction, are distinct between the series. The maximum PSA value reached 134.72 Å^2^ (**2f**), and three groups of compounds were identified with PSA values of 79.67 Å^2^ (**1a**–**d**), 88.9 Å^2^ (**1g**, **2a**–**e** and **2g**–**h**), and 99.9 to 114.2 Å^2^ (**1e**–**f**, **1h**, **2i**–**j**) (Figure 2). The number of rotatable bonds, a parameter involved in the molecule flexibility, was also measured in both series. Four (21%), five (47.4%), and six (31.6%) rotatable bonds were observed (Figure 2). Thus, reduced molecule flexibility (≤10 rotatable bonds) and low PSA (≤140 Å^2^) point to good oral bioavailability of the compounds.

Two main clusters of compounds share similar physicochemical properties. As expected, because of similarity in chemical structure, compounds **1a**–**d** overlap in the same chemical space, with C2 positioned close to this group. High values of HBA and PSA detached compounds **1e**, **1h 1i**, **2f**, **2i,** and **2j** from the selective compounds (**2d**, **1g,** and **C2**) in the chemical space. Data dimension reduction was obtained by principal component analysis (PCA) of six relevant physicochemical properties related to Veber and Lipinski rules. Compounds **2a**–**e**, **2h**, **1f,** and **1g** have a set of similar physicochemical properties and clustered in PCA analysis. Chemical space revealed that the first two PCs account for 75% of variance. HBA account for most (0.54) PC1 impact, followed by PSA (0.51) and rotatable bonds (0.48). HBD had the largest contribution to PC2 (0.24) (Figure 2). Most of modifications in these compound series did not alter significantly the activity against parasite, however the variability of physicochemical properties in the analyzed series by PCA revealed a high influence of hydrogen bond descriptors and PSA, parameters related to compound permeability and polarity, which could impact the activity against intracellular amastigote forms.

The structure-activity relationship (SAR) of both series (**1a**–**i** and **2a**–**j**) was also investigated to consider the activity against intracellular amastigotes (Table 2). To probe the impact of chlorine substitutions on the trypanocidal activity, chlorine atoms were incorporated at the *ortho* (**1c**), *meta* (**1b**), or *para* (**1a**) positions of the R2 benzene ring. Compared to C2 activity (IC_50_ = 9.36 ± 1.9 µM), the addition of chlorine atom to *meta* (IC_50_ = 6.9 ± 1.5 µM) and *para* (IC_50_ = 6.2 ± 0.9 µM) positions slightly improved the biological activity, while chlorine substitution at *ortho* position (IC_50_ = 9.3 ± 0.8 µM) retained the activity of C2, but these analogs have greatly increased cytotoxicity (Table 2). Chlorine atom addition to the *ortho* position of R1 group (**1d**) led to less trypanocidal activity (IC_50_ = 13.5 ± 2.1 µM) but increased cell viability (CC_50_ = 82.1 ± 5.1 µM). Compound **1e**, consisting of *1-(3,5-dichlorophenyl)-1H-1,2,3-triazole* substitution in R2 group, did not improve anti-*T. cruzi* activity (IC_50_ = 9.1 ± 0.7 µM) and enhanced toxicity (CC_50_ = 34.1 ± 7.5 µM). Incorporation of OH to R3 group (**1f**) maintained activity (IC_50_ = 10.1 ± 1.4 µM) but also reduced cell viability (IC_50_ = 41.1 ± 2.1 µM). Replacement of chlorine atom by methoxy group in the *para* position of R2 (**1g**) improved the activity (IC_50_ = 6.7 ± 1.8 µM) and toxicity, reaching a selectivity index value of 14.9, and therefore it is highlighted as the most promising compound in the series. However, the introduction of two (**1h**) or three (**1i**) methoxy groups in R2 resulted in toxicity (24–34 µM) without benefit to trypanocidal activity. The chlorine atom substitution in R1 of **1g** for thiol group (**2j**) led to the loss of trypanocidal activity, reducing by ten-fold its IC_50_ value (Table 2). However, the substitution of the thiol group in R1 of **2j** by the methoxy group (**2i**) increased by approximately four-fold the anti-*T. cruzi* activity (IC_50_ = 15.8 ± 3.2 µM), but reduced by 50% the cell viability (CC_50_ = 44.8 ± 3.3 µM). Although the substitution of the chlorine atom of **1g** for the fluorine atom at the *para* position of the R1 benzene ring (**2e**) resulted in anti-parasitic activity similar to **1g**, compound **2e** showed higher toxicity (CC_50_ = 47.7 ± 3.2 µM). Exchanging the chlorine atom in compound **1g** by the methyl group at the *para* position of the benzene ring (**2d**) reduced compound activity (IC_50_ = 11.9 ± 1.7 µM) but recovered the cell viability (CC_50_ = 141.8 ± 17.6 µM). Substitution of the methyl group at the *ortho* (**2b**) or *meta* (**2c**) position did not alter trypanocidal activity (approximately 12 µM), while its exclusion in compound **2a** increased the activity (IC_50_ = 6.7 ± 1.6 µM), but all these compounds markedly increased toxicity (44–48 µM) compared to **2d** (Table 2). The introduction of nitro (NO_2_) group (**2f**) to R1 instead of thiol group (**2J**) increased ten-fold the biological activity, but the impairment to cell viability is highlighted. Differential anti-*T. cruzi* activity was achieved after addition of propyl (**2g**; IC_50_ = 10.3 ± 2.4 µM) and naphthyl (**2h**; IC_50_ = 22.4 ± 2.2 µM) substituents in R1 but highly increased (2.3-fold) cytotoxicity (Table 2).

### 2.4. Trypanocidal Activity in Mouse Model of Acute Infection

Based on the promising in vitro trypanocidal effects of compound C2, previously identified as a hit compound [29], and the trypanocidal activity of **1g**, we proceed with these compounds for in vivo assay using a murine model of *T. cruzi* acute infection. First, we subjected the mice to five cumulative oral (o.p.) and intraperitoneal (i.p.) doses, 1 h apart, at concentrations of 50 mg/kg and 100 mg/kg of compounds C2 and **1g** for acute toxicity assessment (NOAEL). No toxic side effects, according to the OECD guidelines, were noted up to 48 h post-treatment at the total cumulative dose of 250 mg/kg for both compounds analyzed (data not shown). Additionally, changes in mouse behavioral characteristics were not observed. However, one animal died in the intraperitoneal treatment regimen of **1g** at the cumulative dose of 500 mg/kg.

**Figure 3:**
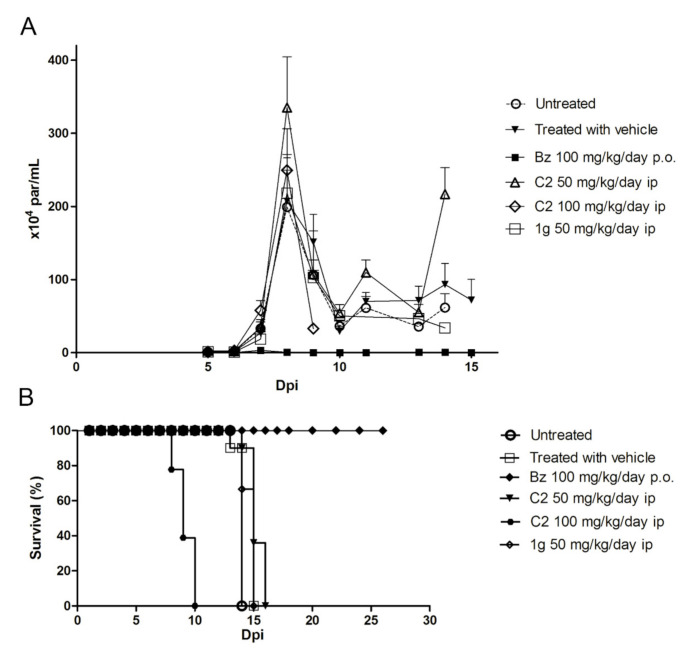
Effect of 1,4-naphthoquinone derivatives (C2 and **1g**) in acute murine model of *T. cruzi* infection. Parasitemia levels (**A**) and survival rate (**B**) of mice intraperitoneally infected with *T. cruzi* trypomastigote forms (Y strain) and submitted to different treatment regimens (*n* = 5 animals/group).

Next, the efficacy of C2 and **1g** was then evaluated in Swiss Webster male mice infected with 10^4^ bloodstream trypomastigotes (Y strain) followed by i.p. treatment with C2 and **1g** for 5 consecutive days after detection of positive parasitemia (5 dpi). Untreated and vehicle-only groups had high levels of parasitemia, as expected for this acute model of *T. cruzi* infection, achieving parasitemia peak at 8 dpi (Figure 3). C2 and **1g** treatment was not efficient in eliminating the parasites. The parasitic load of the C2 and **1g** treated groups remained similar or even slightly higher than the untreated or vehicle-treated control groups (Figure 3). In contrast, the Bz-treated group had undetectable parasitemia by microscopic analysis. Besides not reducing the parasite burden, the compounds did not bring benefits to the animals survival. Treatment with 1,4-naphthoquinones-derived compounds was not able to prevent mortality, as evidenced by Bz treatment (Figure 3). Most animals, untreated and treated with naphthoquinone derivatives (C2 and **1g**) died between 14 and 16 dpi, except for animals treated with 100 mg/kg C2 which died earlier, between 8 and 10 dpi, reaching 100% at the end of treatment (Figure 3). Bz-treated groups survived during all period analyzed (26 days).

## 3. Discussion

The discovery and development of new drugs is a time-consuming (average 10–15 years) and costly ($800 million to $1 billion) process, which makes it a great challenge for the efficient and safe treatment of various diseases [35]. Despite the increased success rate of molecular entity approval in recent years (2017–2018) [36], only 1.65% of the new products for treatment of neglected tropical diseases (NTDs) entered phase I clinical trial [37]. Among the 20 NTDs proposed for control or elimination by 2020, Chagas disease (CD), discovered 110 years ago, has made little progress in finding new drugs effective in treating the disease. The recently released results of the BENDITA study showed the efficacy of Bz in reduced doses (150 mg/kg) and shorter treatment regimen (2 weeks of treatment) in Bolivian patients [17], produced fewer side effects, bringing new hope for individuals with this silent disease. However, screening for new safe drugs are still a priority, because of natural resistance of *T. cruzi* strains to reference drug and its inability to prevent cardiomyopathy, encouraging the search for new hit and lead compounds for CD treatment. In this study, we invested in the optimization of a previously identified hit compound (2-hydroxy-3-phenylsulfanylmethyl-[1,4]-naphthoquinone) and analyzed a small library of 1,4-naphthoquinone analogues for their trypanocidal activity using in vitro and in vivo preclinical assays and computational approaches.

Different strategies, based on C-ring, redox center, and A-ring modifications, have been utilized to develop bioactive naphthoquinoidal derivatives with antiplasmodial, trypanocidal, and leishmanicidal activity [38,39]. Herein, we exploited the synthesis of several lapachol analogues containing [1,2,3]-triazole, thiophenol, alkyl, and naphthalene nucleus. Our findings demonstrated that optimization of compound C2, by addition of chlorine atom in the thiophenol benzene ring, increased the lipophilicity (**1a**–**d**) improving trypanocidal activity compared to C2 and Bz, and also enhancing mammalian cell toxicity. The high lipophilic character of naphthoquinone derivatives containing furane moiety, methoxy group, and aliphatic side chain has been proposed to benefit the trypanocidal activity by improving the compound permeability through the plasma membrane of the parasite [40]. However, the increased toxicity level has been reported to be related to the promiscuity of highly lipophilic compounds (logP >5) which bind with high affinity to nonspecific hydrophobic targets [41]. Alternatively, the insertion of the heterocyclic ring [1,2,3]-triazole into 1,4-naphthoquinone (**1e**) enhanced the activity against trypomastigotes, but not amastigotes, compared to Bz. Optimization of 1,4-naphthoquinones activity by the addition of triazole has generated either promising or completely inactive derivatives against *T. cruzi* [27], suggesting that the position of triazole insertion into quinones or its association with other substitutes modulates its biological activity. High trypanocidal potency has been proposed to be associated with *ortho*- and *para*-quinoidal moieties of [1,2,3]-triazole-coupled naphthoquinones and their electrophilic properties, probably related to high ROS induction [42].

Among the 1,4-naphthoquinone derivatives we highlight compound **1g** in this small library as a promising compound with a better trypanocidal effect. Although the structural changes were minor, only the inclusion of the methoxy group in the benzene ring of the hit compound (C2) has improved the physicochemical properties, including surface polar area, rotatable bonds and hydrogen bond acceptors, making compound **1g** slightly more effective than C2 against trypomastigotes and intracellular amastigotes. It is important to note that merely replacing the chlorine atom with other substituents in series **1(a**–**i)**, including halogens and nitro, propyl, naphthyl groups, affecting the physicochemical properties of the derivatives, induced a reduction in trypanocidal activity and increased toxicity.

SAS map indicated the total number of pairwise matches for all compounds, revealed few compounds with similar structures and different activity in activity in the cliff region. Compound **1g**, the most active compound evidenced by SAR analysis, was identified as a cliff generator on SAS maps (both trypomastigotes and amastigotes) by pairing with at least five distinct derivatives (**2a**, **2b**, **2c**, **2h,** and **2j**), corroborating the relevance of 4-chlorine in the benzene ring (R1). The change of the chlorine atom in R1 by the 4-methyl (**2c**), 5-methyl (**2b**), or thiol group (**2j**) reduced the trypanocidal activity two- to ten-fold. Comparing with C2, the introduction of the methoxy group to benzene ring in R2 improved the lipophilicity and trypanocidal activity of **1g**. In contrast, the other structural changes in R2, by introduction of [1,2,3]-triazole, propyl and naphthyl groups, did not contribute to the improvement of bioactivity. Unfortunately, both compounds C2 and **1g** failed to reduce parasite burden or ensure mouse survival, suggesting that physicochemical parameters still need to be improved for better compound bioavailability and effectiveness. Although numerous naphthoquinone derivatives have been analyzed against *T. cruzi*, few analogues have evolved into preclinical in vivo assays. The 2,3-diphenyl-1,4-naphthoquinone (DPNQ), for instance, has been reported as a potential chemotherapeutic agent against *T. cruzi* because of its trypanocidal activity in phenotypic screening and in murine *T. cruzi* experimental infection [43]. Treatment of C3H/HeN female infected mice with DPNQ reduced two-fold the parasite load and ensured 60% animal survival up to 70 dpi, stimulating compound optimization in an attempt to improve efficacy. In fact, the translational interface between in vitro and in vivo assays as well as preclinical and clinical trials is still a major gap in the development of new drugs, demonstrating the importance of physicochemical and pharmacokinetic properties and the choice of experimental models.

In conclusion, the optimization of compound C2, by introduction of methoxy group in benzene ring (**1g**), moderately improved trypanocidal activity in vitro but highlighted **1g** as cliff generator in SAS map. However, C2 and **1g** were unable to reduce parasite load, and did not prevent mouse mortality. Design and synthesis of a new library of 1,4-naphthoquinones may contribute to the identification of high potent and low toxic drugs for Chagas disease treatment.

## 4. Materials and Methods

### 4.1. Synthetic Compounds

Compounds **1a**–**i** were obtained by multicomponent reaction of lawsone with the appropriate aldehyde to generate the intermediate *o*-quinone methide in situ, followed by nucleophilic addition of a substituted thiol, as previously described by our group [44].

**Figure 4:**
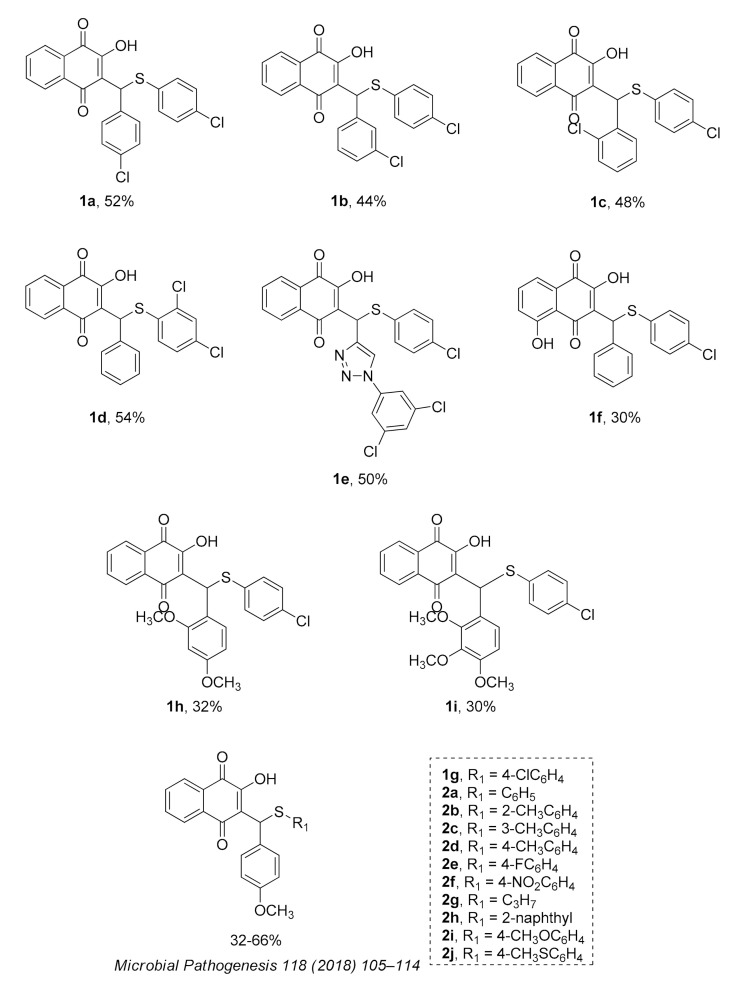
Structure and yields of 2-hydroxy-3-phenylsulfanylmethyl-[1-4]-naphthoquinone derivates.

The reactions were carried out by microwave irradiation (150 °C, 20 min). The results are presented in Figure 4. Naphtoquinones **2a**–**j** were synthetized as previously described [33]. All products were purified by column chromatography using silica gel and were fully characterized by spectroscopic analysis (Supplementary Material).

### 4.2. General Procedure for Preparing **1a**–**i** and **2a**–**j**

A 10 mL microwave tube was loaded with naphthoquinone (2.9 mmol), aldehyde (5.8 mmol), arylthiol (5.8 mmol), and ethanol (5 mL). The mixture was irradiated for 20 min at 150 °C, and the solvent was then evaporated under reduced pressure. The residual was purified by column chromatography on silica gel and eluted with an increasing polarity gradient of hexane and ethyl acetate.

#### 4.2.1. 2-((4-chlorophenyl)((4-chlorophenyl)thio)methyl)-3-hydroxynaphthalene-1,4-dione (**1a**)

The reaction produced the compound **1a** in 52% as a yellow solid of mp 135–136 °C. IR (KBr, cm^−1^): ν 3303, 1668, 1641, 1371, 1251, 720. ^1^H-NMR (CDCl_3_, 500 MHz) δ (*J* in Hz): 8.12 (dd, 1H, *J* = 7.8, 1.0), 8.08 (dd, 1H, *J* = 7.8, 1.0), 7.78 (td, 1H, *J* = 7.8, 1.0), 7.70 (td, 1H, *J* = 7.8, 1.0), 7.68 (s, 1H), 7.58 (d, 2H, *J* = 8.3), 7.29 (d, 2H, *J* = 8.3), 7.27 (d, 2H, *J* = 8.3), 7.21 (d, 2H, *J* = 8.3), 5.81 (s, 1H) ppm. ^13^C NMR (CDCl_3_, 75 MHz): 182.8, 181.3, 152.4, 137.7, 135.5, 134.8, 133.4, 133.3, 133.2, 132.4, 132.3, 129.7, 129.2, 129.1, 128.5, 127.3, 126.4, 122.6, 47.3 ppm. Anal. Calcd. for C_23_H_14_Cl_2_O_3_S: C, 62.59; H, 3.20; (Exp. C, 62.81; H, 3.19).

#### 4.2.2. 2-((3-chlorophenyl)((4-chlorophenyl)thio)methyl)-3-hydroxynaphthalene-1,4-dione (**1b**)

The reaction produced the compound **1b** in 44% as an orange solid of mp 160–161 °C. IR (KBr, cm^−1^): ν 3150, 1672, 1634, 1355, 1278, 1237, 731. ^1^H-NMR (CDCl_3_, 500 MHz) δ (*J* in Hz): 8.13 (dd, 1H, *J* = 7.8, 1.0), 8.08 (dd, 1H, *J* = 7.8, 1.0), 7.79 (td, 1H, *J* = 7.8, 1.0), 7.71 (td, 1H, *J* = 7.8, 1.0), 7.70 (s, 1H), 7.64–7.63 (m, 1H), 7.54–7.52 (m, 1H), 7.31 (d, 2H, *J* = 8.3), 7.25–7.20 (m, 4H), 5.81 (s, 1H) ppm. ^13^C NMR (CDCl_3_, 126 MHz): 182.7, 181.2, 152.4, 141.2, 135.5, 134.7, 134.3, 133.4, 132.4, 132.3, 129.6, 129.2, 129.1, 128.4, 127.6, 127.3, 126.5, 126.4, 122.5, 47.4 ppm. Anal. Calcd. for C_23_H_14_Cl_2_O_3_S: C, 62.59; H, 3.20; (Exp. C, 62.35; H, 3.21).

#### 4.2.3. 2-((2-chlorophenyl)((4-chlorophenyl)thio)methyl)-3-hydroxynaphthalene-1,4-dione (**1c**)

The reaction produced the compound **1c** in 48% as a yellow solid of mp 183–184 °C. IR (KBr, cm^−1^): ν 3327, 1670, 1643, 1364, 1280, 723. ^1^H-NMR (CDCl_3_, 500 MHz) δ (*J* in Hz): 8.27 (dd, 1H, *J* = 7.8, 1.5), 8.13 (dd, 1H, *J* = 7.8, 1.0), 8.07 (dd, 1H, *J* = 7.8, 1.0), 7.78 (td, 1H, *J* = 7.8, 1.0), 7.69 (td, 1H, *J* = 7.8, 1.0), 7.60 (s, 1H), 7.34 (d, 2H, *J* = 8.3), 7.32–7.30 (m, 1H), 7.29 (dd, 1H, *J* = 7.8, 1.5), 7.22–7.20 (m, 3H), 6.21 (s, 1H) ppm. ^13^C NMR (CDCl_3_, 75 MHz): 182.5, 181.5, 153.2, 136.0, 135.6, 134.2, 133.7, 133.5, 133.4, 132.8, 132.5, 132.2, 129.5, 129.4, 128.9, 127.5, 126.7, 126.5, 121.4, 45.7 ppm. Anal. Calcd. for C_23_H_14_Cl_2_O_3_S: C, 62.59; H, 3.20; (Exp. C, 62.76; H, 3.21).

#### 4.2.4. 2-(((2,4-dichlorophenyl)thio)(phenyl)methyl)-3-hydroxynaphthalene-1,4-dione (**1d**)

The reaction produced the compound **1d** in 54% as an orange solid of mp 144–145 °C. IR (KBr, cm^−1^): ν 3326, 1666, 1647, 1349, 1277, 724. ^1^H-NMR (CDCl_3_, 500 MHz) δ (*J* in Hz): 8.13 (dd, 1H, *J* = 7.8, 1.0), 8.07 (dd, 1H, *J* = 7.8, 1.0), 7.78 (td, 1H, *J* = 7.8, 1.0), 7.71–7.70 (m, 1H), 7.70 (td, 1H, *J* = 7.8, 1.0), 7.65 (d, 2H, *J* = 7.3), 7.38 (d, 2H, *J* = 2.0), 7.31 (dd, 2H, *J* = 7.3,7.8), 7.24 (d, 2H, *J* = 8.8), 7.21 (s, 1H), 7.09 (dd, 1H, *J* = 8.8, 2.0), 5.96 (s, 1H) ppm. ^13^C NMR (CDCl_3_, 75 MHz): 183.0, 181.5, 152.9, 138.6, 135.6, 135.5, 134.7, 133.5, 133.0, 132.7, 131.7, 129.7, 129.3, 128.7, 128.4, 127.8, 127.7, 127.5, 126.5, 122.6, 46.2 ppm. Anal. Calcd. for C_23_H_14_Cl_2_O_3_S: C, 62.59; H, 3.20; (Exp. C, 62.56; H, 3.20).

#### 4.2.5. 2-(((4-chlorophenyl)thio)(1-(3,5-dichlorophenyl)-1H-1,2,3-triazol-4-yl)methyl)-3-hydroxynaphthalene-1,4-dione (**1e**)

The reaction produced the compound **1e** in 50% as a red solid of mp 142–143 °C. IR (KBr, cm^−1^): ν 3334, 1673, 1638, 1374, 1258, 740. ^1^H-NMR (DMSO-D_6_, 500 MHz) δ (*J* in Hz): 8.78 (s, 1H), 7.99 (s, 2H), 7.97–7.96 (m, 1H), 7.88–7.87 (m, 1H), 7.76–7.73 (m, 1H), 7.63–7.60 (m, 2H), 7.48 (d, 2H, *J* = 8.4), 7.31 (d, 2H, *J* = 8.4), 6.15 (s, 1H) ppm. ^13^C NMR (DMSO-D_6_, 126 MHz): 183.3, 181.8, 149.1, 138.2, 135.0, 134.9, 134.4, 133.3, 133.2, 132.5, 131.4, 130.9, 130.4, 130.1, 128.4, 127.4, 125.4, 124.8, 121.9, 118.3, 26.1 ppm. Anal. Calcd. for C_25_H_14_Cl_3_N_3_O_3_S: C, 55.32; H, 2.60; N, 7.74; (Exp. C, 55.54; H, 2.61; N, 7.71).

#### 4.2.6. 3-(((4-chlorophenyl)thio)(phenyl)methyl)-2,5-dihydroxynaphthalene-1,4-dione (**1f**)

The reaction produced the compound 1f in 30% as a red solid of mp 164–166 °C. IR (KBr, cm^−1^): ν 3335, 1683, 1616, 1324, 1273, 705. ^1^H-NMR (CDCl_3_, 300 MHz) δ (*J* in Hz): 12.20 (s, 1H), 7.76 (s, 1H), 7.61–7.57 (m, 3H), 7.53–7.48 (m, 1H), 7.29–7.27 (m, 3H), 7.24–7.23 (m, 1H), 7.22–7.15 (m, 4H), 5.80 (s, 1H). ppm. ^13^C NMR (CDCl_3_, 75 MHz): 189.1, 180.8, 161.9, 153.1, 139.0, 135.6, 135.2, 133.4, 132.4, 129.4, 129.3, 128.7, 128.4, 127.7, 127.1, 123.0, 119.8, 114.4, 47.5 ppm. Anal. Calcd. for C_23_H_15_ClO_4_S: C, 65.32; H, 3.58; (Exp. C, 65.09; H, 3.59).

#### 4.2.7. 2-(((4-chlorophenyl)thio)(4-methoxyphenyl)methyl)-3-hydroxynaphthalene-1,4-dione (**1g**)

The reaction produced the compound **1g** in 60% as a yellow solid of mp 122–123 °C. IR (KBr, cm^−1^): ν 3282, 1666, 1648, 1508. ^1^H-NMR (CDCl_3_, 300 MHz) δ (*J* in Hz): 8.11 (dd, 1H, *J* = 7.8, 1.0), 8.06 (dd, 1H, *J* = 7.3, 1.0), 7.76 (td, 1H, *J* = 7.3, 1.0), 7.74 (s, 1H), 7.68 (dt, 1H, J = 7.8, 1.0), 7.56 (d, 2H, *J* = 8.8), 7.30 (d, 2H, *J* = 8.3), 7.19 (d, 2H, *J* = 8.8), 6.84 (d, 2H, *J* = 8.3), 5.84 (s, 1H), 3.77 (s, 3H) ppm. ^13^C NMR (CDCl_3_, 75 MHz): 183.0, 181.4, 158.8, 152.2, 135.3, 133.2, 132.9, 132.4, 132.0, 131.1, 129.4, 129.1, 129.0, 127.2, 126.2, 123.1, 113.7, 55.2, 47.1 ppm. Anal. Calcd. for C_24_H_17_ClO_4_S: C, 65.98; H, 3.92; (Exp. C, 65.74; H, 3.91).

#### 2.4.8. 2-(((4-chlorophenyl)thio)(2,4-dimethoxyphenyl)methyl)-3-hydroxynaphthalene-1,4-dione (**1h**)

The reaction produced the compound **1h** in 35% as a brown solid of mp 70–71 °C. IR (KBr, cm^−1^): ν 3331, 1648, 1586, 1258, 1094, 724. ^1^H-NMR (DMSO-D_6_, 300 MHz) δ (*J* in Hz): 8.00–7,96 (m, 3H), 7.83 (td, 2H, *J* = 7.4, 1.6), 7.78 (dd, 1H, *J* = 7.4, 1.6), 7.73 (d, 2H, *J* = 8.4), 6.51 (d, 1H, *J* = 2.5), 6.48–6.46 (m, 2H), 6.11 (s, 1H), 3.72 (s, 3H), 3.63 (s, 3H) ppm. ^13^C NMR (DMSO-D_6_, 75 MHz): 182.3, 180.8, 159.5, 157.2, 154.9, 136.3, 134.5, 132.9, 132.1, 131.6, 130.8, 129.6, 129.1, 128.5, 125.7, 125.4, 123.7, 122.6, 104.6, 98.1, 55.4, 54.9, 40.9 ppm. Anal. Calcd. for C_25_H_19_ClO_5_S: C, 64.31; H, 4.10; (Exp. C, 64.58; H, 4,09).

#### 4.2.9. 2-(((4-chlorophenyl)thio)(2,3,4-trimethoxyphenyl)methyl)-3-hydroxynaphthalene-1,4-dione (**1i**)

The reaction produced the compound **1i** in 30% as a yellow solid of mp 116–117 °C. IR (KBr, cm^−1^): ν 1671, 1641, 1589, 1289, 1081, 720. ^1^H-NMR (CDCl_3_, 500 MHz) δ (*J* in Hz): 8.13–8.11 (m, 1H), 8.07–8.05 (m, 1H), 7.77–7.74 (m, 2H), 7.72–7.70 (m, 1H), 7.69–7.66 (m, 1H), 7.34 (d, 2H, *J* = 8.2), 7.19 (d, 2H, *J* = 8.2), 6.70–6.68 (m, 1H), 6.16 (s, 1H), 3.85 (s, 3H), 3.80 (s, 3H), 3.78 (s, 3H) ppm. ^13^C NMR (DMSO-D_6_, 126 MHz): 182.4, 180.8, 155.0, 152.4, 150.5, 136.0, 134.5, 133.0, 131.6, 130.9, 130.8, 129.6, 128.6, 125.8, 125.5, 124.6, 122.7, 108.1, 60.2, 55.7, 41.1 ppm. Anal. Calcd. for C_26_H_21_ClO_6_S: C, 62.84; H, 4.26; (Exp. C, 62.79; H, 4.25).

#### 4.2.10. 2-Hydroxy-3-((4-methoxyphenyl)(phenylthio)methyl)naphthalene-1,4-dione (**2a**)

The reaction produced the compound **2a** in 66% as an orange solid of mp 49–51 °C. IR (KBr, cm^−1^) ν 3338, 1669, 1645, 1509. ^1^H-NMR (CDCl_3_, 300 MHz) δ (*J* in Hz): 8.12 (dd, 1H, *J* = 7.7, 1.1), 8.06 (dd, 1H, *J* = 7.7, 1.1), 7.76 (dt, 1H, *J* = 7.7, 1.1), 7.72 (s, 1H), 7.68 (dt, 1H, *J* = 7.7, 1.1), 7.58 (d, 2H, *J* = 8.8), 7.39–7.37 (m, 2H), 7.22 (t, 2H, *J* = 7.7), 7.18 (t, 1H, *J* = 7.7), 6.84 (d, 2H, *J* = 8.8), 5.91 (s, 1H), 3.78 (s, 3H) ppm. ^13^C NMR (CDCl_3_, 75 MHz): 182.9, 181.3, 158.7, 152.3, 136.7, 135.1, 133.0, 132.4, 131.4, 130.4, 129.4, 129.1, 128.8, 127.0, 126.7, 126.1, 123.4, 113.6, 55.1, 46.7 ppm. Anal. Calcd. for C_24_H_18_O_4_S: C, 71.62; H, 4.51; (Exp. C, 71.47; H, 4.52).

#### 4.2.11. 2-Hydroxy-3-((4-methoxyphenyl)(o-tolylthio)methyl) naphthalene-1,4-dione (**2b**)

The reaction produced the compound **2b** in 59% as an orange solid of mp 54–55 °C. IR (KBr, cm^−1^) ν 3335, 1667, 1509, 1645. ^1^H-NMR (CDCl_3_, 300 MHz) δ (*J* in Hz): 8.11 (dd, 1H, *J* = 7.3, 1.0), 8.06 (dd, 1H, *J* = 7.8, 1.0), 7.75 (dt, 1H, *J* = 7.8, 1.0), 7.74 (s, 1H), 7.68 (dt, 1H, *J* = 7.3, 1.0), 7.58 (d, 2H, *J* = 8.8), 7.29 (dd, 1H, *J* = 7.3, 1.0), 7.15 (d, 1H, *J* = 6.8), 7.10–7.04 (m, 2H), 6.83 (d, 2H, *J* = 8.8), 5.84 (s, 1H), 3.77 (s, 3H), 2.41 (s, 3H) ppm. ^13^C NMR (CDCl_3_, 75 MHz): 181.5, 152.3, 138.7, 135.7, 135.2, 133.1, 132.5, 131.3, 130.7, 130.1, 129.5, 129.1, 127.2, 126.8, 126.5, 126.2, 123.5, 113.7, 55.2, 46.0, 20.6 ppm. Anal. Calcd. for C_25_H_20_O_4_S: C, 72.09; H, 4.84; (Exp. C, 71.98; H, 4.82).

#### 4.2.12. 2-Hydroxy-3-((4-methoxyphenyl)(m-tolylthio)methyl)naphthalene-1,4-dione (**2c**)

The reaction produced the compound **2c** in 65% as a dark red solid of mp 45–47 °C. IR (KBr, cm^−1^) ν 3346, 1668, 1646, 1509. ^1^H-NMR (CDCl_3_, 300 MHz) δ (*J* in Hz): 8.11 (dd, 1H, *J* = 7.7, 1.1), 8.06 (dd, 1H, *J* = 7.7, 1.1), 7.75 (dt, 1H, *J* = 7.7, 1.1), 7.76 (s, 1H), 7.67 (dt, 1H, *J* = 7.7, 1.1), 7.56 (d, 2H, *J* = 8.8), 7.20 (s, 1H), 7.17 (d, 1H, *J* = 8.2), 7.11 (t, 1H, *J* = 7.7), 6.98 (d, 1H, *J* = 7.7), 6.84 (d, 2H, *J* = 8.8), 5.90 (s, 1H), 3.77 (s, 3H), 2.26 (s, 3H) ppm. ^13^C NMR (CDCl_3_, 75 MHz): 183.0, 181.5, 158.7, 152.3, 138.6, 136.4, 135.2, 133.1, 132.5, 131.5, 131.1, 129.4, 129.2, 128.7, 127.7, 127.4, 127.1, 126.2, 123.5, 113.7, 55.2, 46.7, 21.3 ppm. Anal. Calcd. for C_25_H_20_O_4_S: C, 72.09; H, 4.84; (Exp. C, 72.01; H, 4.85).

#### 4.2.13. 2-Hydroxy-3-((4-methoxyphenyl)(p-tolylthio)methyl) naphthalene-1,4-dione (**2d**)

The reaction produced the compound **2d** in 58% as a dark red solid of mp 55–56 °C. IR (KBr, cm^−1^) ν 3391, 1646, 1509. ^1^H-NMR (CDCl_3_, 300 MHz) δ (*J* in Hz): 8.10 (dd, 1H, *J* = 7.6, 1.2), 8.05 (dd, 1H, *J* = 7.6, 1.2), 7.77–7.72 (m, 2H), 7.67 (dt, 1H, *J* = 7.6, 1.2), 7.56 (d, 2H, *J* = 8.8), 7.29 (d, 2H, *J* = 8.2), 7.03 (d, 2H, *J* = 8.2), 6.83 (d, 2H, *J* = 8.8), 5.83 (s, 1H), 3.77 (s, 3H), 2.27 (s, 1H) ppm. ^13^C NMR (CDCl_3_, 75 MHz): 183.0, 181.5, 158.7, 152.3, 137.1, 135.2, 133.1, 132.8, 132.5, 131.5, 131.4, 129.7, 129.5, 129.2, 127.2, 126.2, 123.6, 113.7, 55.2, 47.5, 21.0 ppm. Anal. Calcd. for C_25_H_20_O_4_S: C, 72.09; H, 4.84; (Exp. C, 71.85; H, 4.84).

#### 4.2.14. 2-(((4-fluorophenyl)thio)(4-methoxyphenyl)methyl)-3-hydroxynaphthalene-1,4-dione (**2e**)

The reaction produced the compound **2e** in 63% as a yellow solid of mp 132–134 °C. IR (KBr, cm^−1^) ν 3255, 1665, 1649, 1509. ^1^H-NMR (CDCl_3_, 300 MHz) δ (*J* in Hz): 8.11 (dd, 1H, *J* = 7.8, 1.0), 8.06 (dd, 1H, *J* = 7.8, 1.0), 7.76 (dt, 1H, *J* = 7.3, 1.0), 7.71 (s, 1H), 7.68 (dt, 1H, *J* = 7.3, 1.0), 7.55 (d, 2H, *J* = 8.3), 7.39–7.36 (m, 2H), 6.92 (t, 2H, *J* = 8.8), 6.84 (d, 2H, *J* = 8.3), 5.77 (s, 1H), 3.77 (s, 3H) ppm. ^13^C NMR (CDCl_3_, 75 MHz): 183.0, 181.4, 161.5, 158.8, 152.3, 135.2, 133.9, 133.2, 132.5, 131.5, 131.3, 129.5, 129.1, 127.2, 126.2, 123.4, 115.9, 113.7, 55.2, 40.1 ppm. Anal. Calcd. for C_24_H_17_FO_4_S: C, 68.56; H, 4.08; (Exp. C, 68.34; H, 4.09).

#### 4.2.15. 2-Hydroxy-3-((4-methoxyphenyl)((4-nitrophenyl)thio)methyl)naphthalene-1,4-dione (**2f**)

The reaction produced the compound **2f** in 32% as a light brown solid of mp 63–64 °C. IR (KBr, cm^−1^) ν 3369, 1672, 1645, 1510, 1338. ^1^H-NMR (CDCl_3_, 300 MHz) δ (*J* in Hz): 8.14 (dd, 1H, *J* = 7.9, 0.6), 8.07–8.05 (m, 3H), 7.78 (dt, 1H, *J* = 7.9, 1.2), 7,70 (dt, 1H, *J* = 7.9, 0.6), 7.61–7.59 (m, 2H), 7.38–7.36 (m, 2H), 6.86–6.85 (m, 2H),6.09 (s, 1H), 3.77 (s, 3H) ppm. ^13^C NMR (CDCl_3_, 75 MHz): 182.9, 181.2, 159.1, 152.5, 147.5, 145.4, 135.5, 133.4, 132.3, 130.0, 129.3, 129.0, 127.2, 126.4, 124.0, 122.1, 114.0, 55.2, 44.6 ppm. Anal. Calcd. for C_24_H_17_NO_6_S: C, 64.42; H, 3.83; N, 3.13; (Exp. C, 64.40; H, 3.84; N, 3.12).

#### 4.2.16. 2-Hydroxy-3-((4-methoxyphenyl)(propylthio)methyl)naphthalene-1,4-dione (**2g**) 

The reaction produced the compound **2g** in 51% as a red solid of mp 32–33 °C. IR (KBr, cm^-1^) ν 3306, 1654, 1640, 1592. ^1^H-NMR (CDCl_3_, 300 MHz) δ (*J* in Hz): 8.11 (dd, 1H, *J* = 7.8, 1.0), 8.06 (dd, 1H, *J* = 7.8, 1.0), 8.02 (s, 1H), 7.74 (dt, 1H, *J* = 7.3, 1.0), 7,67 (dt, 1H, *J* = 7.3, 1.0), 7.54 (d, 2H, *J* = 8.8), 6.83 (d, 2H, *J* = 8.8), 5.52 (s, 1H), 3.77 (s, 3H), 2.58–2.54 (m, 2H), 1.65–1.63 (m, 2H), 0.96 (t, 3H, *J* = 7.3) ppm. ^13^C NMR (CDCl_3_, 75 MHz): 183.2, 181.4, 164.4, 158.7, 152.8, 135.6, 133.1, 132.5, 131.5, 129.5, 127.1, 126.1, 123.6, 113.7, 55.5, 43.0, 35.4, 22.7, 13.5 ppm. Anal. Calcd. for C_21_H_20_O_4_S: C, 68.46; H, 5.47; (Exp. C, 68.34; H, 5.49).

#### 4.2.17. 2-Hydroxy-3-((4-methoxyphenyl)(naphthalen-2-ylthio)methyl) naphthalene-1,4-dione (**2h**)

The reaction produced the compound **2h** in 35% as a red solid of mp 56–57 °C. IR (KBr, cm^−1^) ν 3357, 1669, 1646, 1509. ^1^H-NMR (CDCl_3_, 300 MHz) δ (*J* in Hz): 8.12 (dd, 1H, *J* = 7.8, 1.0), 8.05 (dd, 1H, *J* = 7.3, 1.0), 7.83–7.82 (m, 1H), 7.77–7.73 (m, 3H), 7.72–7.70 (m, 1H), 7.69–7.66 (m, 2H), 7.62 (d, 2H, *J* = 8.8), 7.48–7.46 (m, 1H), 7.43–7.41 (m, 2H), 6.85 (d, 2H, *J* = 8.8), 6.04 (s, 1H), 3.77 (s, 3H) ppm. ^13^C NMR (CDCl_3_, 75 MHz): 183.0, 181.3, 158.7, 152.3, 135.1, 134.2, 133.5, 133.0, 132.4, 132.0, 131.4, 129.5, 129.1, 128.7, 128.4, 128.1, 127.6, 127.3, 127.1, 126.3, 126.1, 125.8, 123.3, 113.7, 55.2, 44.6 ppm. Anal. Calcd. for C_28_H_20_O_4_S: C, 74.32; H, 4.45; (Exp. C, 74.23; H, 4.46).

#### 4.2.18. 2-Hydroxy-3-((4-methoxyphenyl)((4-methoxyphenyl)thio)methyl)naphthalene-1,4-dione (**2i**)

The reaction produced the compound **2i** in 50% as a light brown solid of mp 104–106 °C. IR (KBr, cm^-1^) ν 3329, 1664, 1649, 1508. ^1^H-NMR (CDCl_3_, 300 MHz) δ (*J* in Hz): 8.11–8.08 (m, 1H), 8.06 (dd, 1H, *J* = 7.6, 1.2), 7.77–7.72 (m, 2H), 7.67 (dt, 1H, *J* = 7.6, 1.2), 7.54 (d, 2H, *J* = 8.8), 7.35 (d, 2H, *J* = 8.8), 6.83 (d, 2H, *J* = 8.8), 6.76 (d, 2H, *J* = 8.8), 5.72 (s, 1H), 3.77 (s, 3H), 3.75 (s, 3H) ppm. ^13^C NMR (CDCl_3_, 75 MHz): 183.0, 181.5, 159.4, 158.7, 152.3, 135.1, 134.4, 133.1, 132.5, 131.6, 129.6, 129.2, 127.1, 126.7, 126.1, 123.6, 114.5, 113.6, 55.2, 48.7 ppm. Anal. Calcd. for C_25_H_20_O_5_S: C, 69.43; H, 4.66; (Exp. C, 69.38; H, 4.66).

#### 4.2.19. 2-hydroxy-3-((4-methoxyphenyl)((4-(methylthio)phenyl)thio)methyl)naphthalene-1,4-dione (**2j**)

The reaction produced the compound **2j** in 36% as a dark brown solid of mp 45–47 °C. IR (KBr, cm^−1^) ν 3350, 1668, 1645, 1509. ^1^H-NMR (CDCl_3_, 300 MHz) δ (*J* in Hz): 8.11 (dd, 1H, *J* = 7.6, 1.2), 8.06 (dd, 1H, *J* = 7.6, 1.2), 7.78–7.72 (m, 2H), 7.67 (dt, 1H, *J* = 7.6, 1.2), 7.56 (d, 2H, *J* = 8.8), 7.30 (d, 2H, *J* = 8.2), 7.10 (d, 2H, *J* = 8.2), 6.83 (d, 2H, *J* = 8.8), 5.82 (s, 1H), 3.77 (s, 3H), 2.42 (s, 3H) ppm. ^13^C NMR (CDCl_3_, 75 MHz): 183.0, 181.4, 158.8, 152.3, 137.8, 135.2, 133.1, 132.8, 132.5, 131.7, 131.4, 129.5, 129.1, 127.2, 126.9, 126.2, 123.4, 113.7, 55.2, 47.5, 15.7 ppm. Anal. Calcd. for C_25_H_20_O_4_S_2_: C, 66.94; H, 4.49; (Exp. C, 66.68; H, 4.51).

### 4.3. Cell Culture

Confluent cultures of Vero cells (ATCC^®^ CCL 81™) were dissociated with trypsin-EDTA solution (0.025%) and cultivated in RPMI-1640 medium supplemented with 10% fetal bovine serum (FBS). The cultures were maintained at 37 °C in humidified atmosphere of 5% CO_2_.

### 4.4. Parasites

*T. cruzi* Dm28c-Luc clone, genetically modified to express firefly luciferase, and Y strain were used in the drug screening assay. Vero cells were infected with *T. cruzi*, Dm28c-Luc or Y strain, in a 10:1 parasites/host cell ratio and maintained at 37 °C in humidified atmosphere of 5% CO_2_. Trypomastigotes were harvested from the infected culture supernatant on the 4th day post-infection (dpi) followed by quantification of the number of parasites/mL in Neubauer chamber.

### 4.5. Cytotoxicity In Vitro Assay

To evaluate the toxic effects of the 1,4-naphthoquinone derivatives on mammalian cells, Vero cells were seeded at a density of 1.5 × 10^4^ cells/well in 96-well white culture plates. Twenty-four hours later, the cell cultures were incubated for 72 h at 37 °C with 1,4-naphthoquinone derivatives (series **1**(**a***–***i**) and **2**(**a**–**j**)) and Bz (500–1.9 µM) diluted in RPMI medium supplemented with 10% FBS. After incubation, the cell viability, measured by ATP level, was assessed by adding 20 µL/well of the CellTiter Glo (Promega Corporation, Madison, WI, EUA) solution. The luminescent signal was read on the FlexStation 3 reader (Molecular Devices, Sunnyvale, CA, USA). The concentration of compound that reduces 50% of mammalian cell viability (CC_50_) was determined by linear regression. All treatments and controls were performed at low concentration (≤1%) of dimethyl sulfoxide (DMSO). At least three independent assays were performed in duplicate.

### 4.6. Trypanocidal Activity

Screening of the compounds was performed against trypomastigotes and intracellular amastigote forms. Trypomastigotes (1 × 10^6^ parasites/well), Dm28c-Luc clone and Y strain, were incubated for 24 h at 37 °C with the 1,4 naphthoquinone derivatives and Bz (0.41–100 μM) and their viability determined by the activity of the luciferase enzyme, after addition of luciferin substrate (300 μg/mL), or CellTiter Glo. The luminescent signal was detected on the FlexStation 3 reader.

The effect of the compounds against intracellular amastigotes was screened in 72 h *T. cruzi*-infected Vero cells. Briefly, Vero cells seeded (1.5 × 10^4^ cells/well) on 96-well white plate infected with trypomastigotes (Dm28c-Luc) at a 10:1 multiplicity of parasites/cell. After 24 h of infection, the cultures were washed with PBS and then, treated for 72 h at 37 °C with naphthoquinone derivatives and Bz (0.41–100 µM). After treatment, luciferin (300 µg/mL) was added to the culture and the viability of the parasites was assessed by reading the luminescent signal using the FlexStation 3 reader. Controls were also performed in no-toxic concentrations of DMSO (≤1%). The concentration that reduces the number of viable parasites by 50% (IC_50_) or 90% (IC_90_) was calculated by linear regression.

### 4.7. In Silico Analysis

Molecular structure of compounds (SMILES code) and pIC50 (-log IC_50_) values were inserted in Datawarrior software version 5.0 to explore the chemical space, physicochemical properties, and structure activity-relationship (SAR) [45]. For principal component analysis (PCA), six properties related to Veber and Lipinski rules were applied for each compound. The activity landscape and the structure-activity similarity (SAS) maps were analyzed using Activity Landscape Plotter, an online open web platform (https://www.difacquim.com/d-tools/). MACCS molecular fingerprints were used in SAS map analysis and the threshold for structural similarity were set to 0.8.

### 4.8. Mouse Acute Toxicity

To establish the no-observed-adverse-effect level (NOAEL), increasing doses of the 1,4 naphthoquinone derivatives were orally (o.p.) and intraperitoneally (i.p.) administered into male Swiss mice (21 to 24 g; *n* = 2 for each group). Treated animals were separated into two groups: (1) 5 consecutive doses of 50 mg/kg and (2) 100 mg/kg every 1h, totaling a cumulative dose of 250 mg/kg and 500 mg/kg, respectively. Untreated and vehicle treated animals (PBS with 3% Tween-80) were used as control groups. Mice were inspected for toxic and sub-toxic symptoms according to Organization for Economic Co-operation and Development (OECD) guidelines [46].

### 4.9. In Vivo Experimental T. cruzi Infection

Male Swiss Webster mice (18 g) were obtained from the Institute of Science and Technology in Bio-models of the Oswaldo Cruz Foundation, Rio de Janeiro, Brazil. Mice were housed in groups of 5 animals per cage in a ventilated cabinet under controlled temperature (22 ± 1 °C), 55% ± 5% humidity and a 12 h light-dark light cycle. The animals received water and an ad libitum feeding regimen. Mice were intraperitoneally (i.p.) infected with bloodstream trypomastigotes of *T. cruzi* Y strain (10^4^ parasites/animal) and parasitemia was daily evaluated from the 4th dpi. Animals were distributed into ten groups: (1) uninfected and untreated, (2) uninfected and treated with C2 (50 mg/kg); (3) uninfected and treated with C2 (100 mg/kg); (4) uninfected and treated with **1g** (50 mg/kg), (5) *T. cruzi*-infected and untreated, (6) *T. cruzi*-infected and treated i.p. with vehicle, (7) *T. cruzi*-infected and treated i.p. with C2 50 mg/kg/day, (8) *T. cruzi* infected and treated i.p. with C2 100 mg/kg/day, (9) *T. cruzi*-infected and treated i.p. with **1g** 50 mg/kg/day, and (10) *T. cruzi*-infected and treated orally with Bz 100 mg/kg/day. Daily treatment (0.1 mL i.p. dose) started at 5 days post-infection (dpi) with positive parasitemia and followed up to 10 dpi.

Parasitemia was estimated by Pizzy-Brener method [47]. Briefly, animals parasitemia were determined by microscopic quantification of fresh blood (5 µL) collected from animals’ caudal vein. Mortality was daily evaluated up to 26 days and expressed in survival rates. All animal procedures were previously approved by the Oswaldo Cruz Institute Ethical Committee for the Use of Animals (License L15/17).

## Figures and Tables

**Table 1:** Cytotoxicity and trypanocidal effect of 1,4-naphthoquinone derivatives against trypomastigote forms of T. cruzi.

Compounds	Trypanocidal Effect IC_50_Trypomastigotes (Mean ± SD μM)				Vero Cells Toxicity CC_50_ (Mean ± SD μM)	SI * Trypomastigotes	
	Dm28c-Luc		Y Strain			Dm28c-Luc	Y
	IC_50_	IC_90_	IC_50_	IC_90_			
**1a**	8.9 ± 0.6	25.1 ± 1.6	3.1 ± 0.6	5.8 ± 0.9	43.6 ± 2.3	4.8	15.0
**1b**	9.8 ± 0.5	30.8 ± 2.9	3.6 ± 1.6	8.1 ± 1.3	42.1 ± 1.9	4.2	11.7
**1c**	8.6 ± 1.4	29.6 ± 5.4	4.3 ± 3.2	7.3 ± 2.2	44.1 ± 2.3	5.1	10.2
**1d**	9.3 ± 1.1	23.3 ± 2.1	2.8 ± 0.6	5.8 ± 1.1	82.1 ± 5.1	8.9	29.3
**1e**	4.5 ± 0.8	16.5 ± 2.8	4.7 ± 1.9	5.5 ± 0.9	34.1 ± 2.9	7.5	7.2
**1f**	9.8 ± 0.7	28.1 ± 1.7	22.3 ± 0.3	10.9 ± 2.1	41.1 ± 2.1	4.2	1.8
**1g**	8.1 ± 0.6	19.3 ± 3.4	10.2 ± 0.5	16.8 ± 2.5	99.9 ± 12.5	12.3	9.8
**1h**	15.4 ± 4.6	15.4 ± 4.6	22.9 ± 7.1	31.9 ± 6.1	24.4 ± 2.6	1.5	1.1
**1i**	21.5 ± 2.9	21.5 ± 2.9	30.1 ± 2.3	38.9 ± 4.3	34.2 ± 3.4	1.6	1.1
**2a**	27.4 ± 2.9	32.1 ± 1.6	31.2 ± 4.3	38.9 ± 5.3	45.1 ± 1.9	1.6	1.4
**2b**	25.9 ± 2.2	31.8 ± 0.9	27.9 ± 4.1	37.9 ± 6.1	44.4 ± 3.4	1.7	1.5
**2c**	52.3 ± 6.4	84.6 ± 11.4	26.2 ± 4.8	32.8 ± 5.1	48.9 ± 4.7	0.9	1.8
**2d**	24.3 ± 3.4	82.2 ± 7.7	31.1 ± 1.8	49.7 ± 7.1	141.8 ± 17.6	5.4	4.5
**2e**	24.4 ± 4.4	31.4 ± 4.4	20.3 ± 4.1	32.3 ± 5.1	47.7 ± 3.2	1.9	2.3
**2f**	6.5 ± 3.1	11.5 ± 3.1	12.5 ± 1.6	19.9 ± 3.6	40.6 ± 2.9	6.2	3.2
**2g**	13.7 ± 4.1	28.7 ± 5.1	12.7 ± 3.9	23.7 ± 4.1	47.7 ± 5.2	3.5	3.7
**2h**	21.7 ± 4.7	39.7 ± 5.7	24.5 ± 3.6	34.5 ± 4.6	63.4 ± 7.1	2.9	2.5
**2i**	24.1 ± 2.7	44.9 ± 6.7	10.6 ± 0.4	31.6 ± 5.4	44.8 ± 3.3	1.8	4.2
**2j**	52.7 ± 12.6	>100	89.6 ± 5.4	>100	93.5 ± 6.6	1.7	1.1
**C2** ^#^	21.3 ± 1.9	31.6 ± 0.4	10.1 ± 1.0	27.8 ± 4.6	178.9 ± 6.2	8.4	17.6
Bz	17.5 ± 3.3	>100	14.3 ± 3.2	>100	>500	>28.6	>34.9

Mean values of three independent experiments ± standard deviations; IC_50_ and IC_90_: concentration producing 50% and 90% inhibitory effect on *T. cruzi* trypomastigote forms, respectively; CC_50_: concentration that reduces 50% of Vero cell viability. * SI = CC_50_ Vero cells/IC_50_ trypomastigote forms of *T. cruzi*. C2—Hit compound (^#^ data from Lara et al., 2018 [29]).

**Table 2:** Trypanocidal activity and structure activity relationship (SAR) of 1,4- naphthoquinone derivatives. The effect of changes in R-groups against intracellular amastigotes of *T. cruzi* (Dm28c-Luc clone).

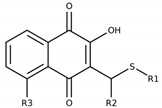							
**Compounds**	**R1**	**R2**	**R3**	**IC_50_ Ama (µM)**	**IC_90_ Ama (µM)**	**CC_50_ Vero (µM)**	**SI**
**1a**	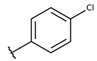	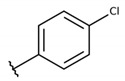	-	6.2 ± 0.9	12.1 ± 1.3	43.6 ± 2.3	7.0
**1b**	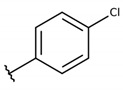	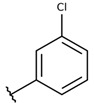	-	6.9 ± 1.5	12.7 ± 1.8	42.1 ± 1.9	6.1
**1c**	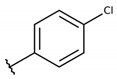	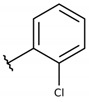	-	9.3 ± 0.8	23.1 ± 3.3	44.1 ± 2.3	4.7
**1d**	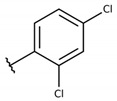	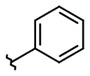	-	13.5 ± 2.1	31.1 ± 3.1	82.1 ± 5.1	6.2
**1e**	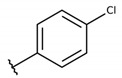	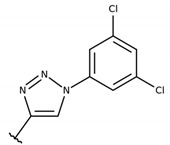	-	9.1 ± 0.7	27.9 ± 3.9	34.1 ± 7.5	3.7
**1f**	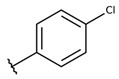	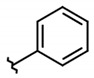	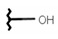	10.1 ± 1.4	29.1 ± 2.1	41.1 ± 2.1	4.1
**1g**	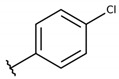	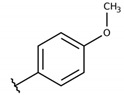	-	6.7 ± 1.8	11.4 ± 2.1	99.5 ± 12.5	14.9
**1h**	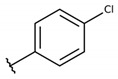	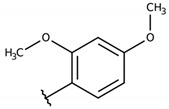	-	12.1 ± 3.9	19.4 ± 3.6	24.4 ± 2.6	2.0
**1i**	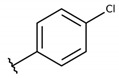	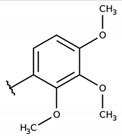	-	15.9 ± 3.1	27.2 ± 3.4	34.2 ± 3.4	2.1
**2a**	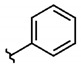	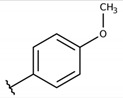	-	6.7 ± 1.6	25.3 ± 1.9	45.1 ± 1.9	6.7
**2b**	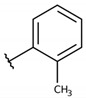	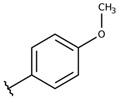	-	11.3 ± 1.1	27.4 ± 2.4	44.4 ± 3.4	3.9
**2c**	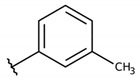	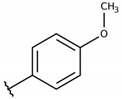	-	12.6 ± 2.4	30.9 ± 4.7	48.9 ± 4.7	3.8
**2d**	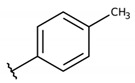	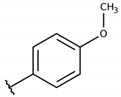	-	11.9 ± 1.7	28.8 ± 4.6	141.8 ± 17.6	11.9
**2e**	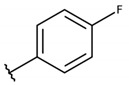	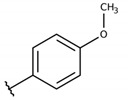	-	6.8 ± 1.5	19.7 ± 3.2	47.7 ± 3.2	7.0
**2f**	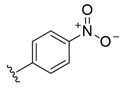	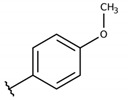	-	5.7 ± 1.4	76.6 ± 4.9	40.6 ± 2.9	7.1
**2g**	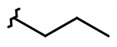	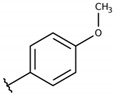	-	10.3 ± 2.4	37.7 ± 4.1	47.7 ± 5.2	4.6
**2h**	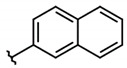	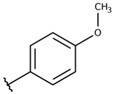	-	22.4 ± 2.2	31.5 ± 6.1	63.4 ± 7.1	2.8
**2i**	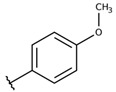	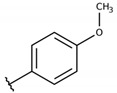	-	15.8 ± 3.2	34.8 ± 5.3	44.8 ± 3.3	2.8
**2j**	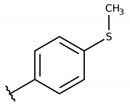	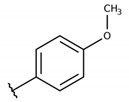	-	60.2 ± 9.8	>100	93.5 ± 6.6	1.5
**Bz**	-	-	-	1.4 ± 0.4	7.9 ± 1.2	>500	>357
**C2** *	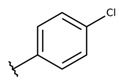	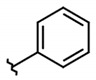	-	9.36 ± 1.90	22.6 ± 0.6	178.90 ± 6.18	19.1

Vero cells infected with *T. cruzi*, Dm28c-Luc clone (expressing luciferase gene), and treated for 72 h with naphthoquinones derivatives. The data represent the mean and standard deviation of three independent experiments. IC_50_: concentration producing 50% inhibitory effect of intracellular amastigote forms. CC_50_: concentration that reduces 50% of Vero cell viability. * SI = CC_50_ Vero cells/IC_50_ of amastigote forms of *T. cruzi*. C2—Hit compound (* data from Lara et al. 2018 [29]).

## Supplementary Materials

The following are available online.

## Sample Availability

Not available.
