# Supplementary material for: Optimization of 1,4-Naphthoquinone Hit Compound: A Computational, Phenotypic, and In Vivo Screening against Trypanosoma cruzi
Source: Molecules. 2021 Jan 15;26(2):423. doi: 10.3390/molecules26020423 (PMC7829778; doi:10.3390/molecules26020423)
Supplement: Supplementary file 1 [file molecules-26-00423-s001.pdf]

SUPPORTING INFORMATION

Optimization of 1,4-naphthoquinone hit compound: a computational, phenotypic and in vivo screening against *Trypanosoma cruzi*

L. S. Lara[a], G. C. Lechuga[a], C. S. Moreira[b], T. B. Santos[b], V. F. Ferreira[b], D. R. da Rocha[b] and M.C.S. Pereira[a]

[a] Laboratório de Ultraestrutura Celular, Instituto Oswaldo Cruz, Fiocruz, Av. Brasil 4365 Manguinhos, 21040-900 Rio de Janeiro, RJ, Brazil.

[b]Departamento de Química Orgânica, Instituto de Química, Universidade Federal Fluminense, Rua Outeiro São João Batista, 24020-141 Niterói, Rio de Janeiro, Brazil.

Contents

1. Analysis for Compound **1a** II
2. Analysis for Compound **1b** III
3. Analysis for Compound **1c** V
4. Analysis for Compound **1d** VI
5. Analysis for Compound **1e** VIII
6. Analysis for Compound **1f** IX
7. Analysis for Compound **1h** XI
8. Analysis for Compound **1i** XII

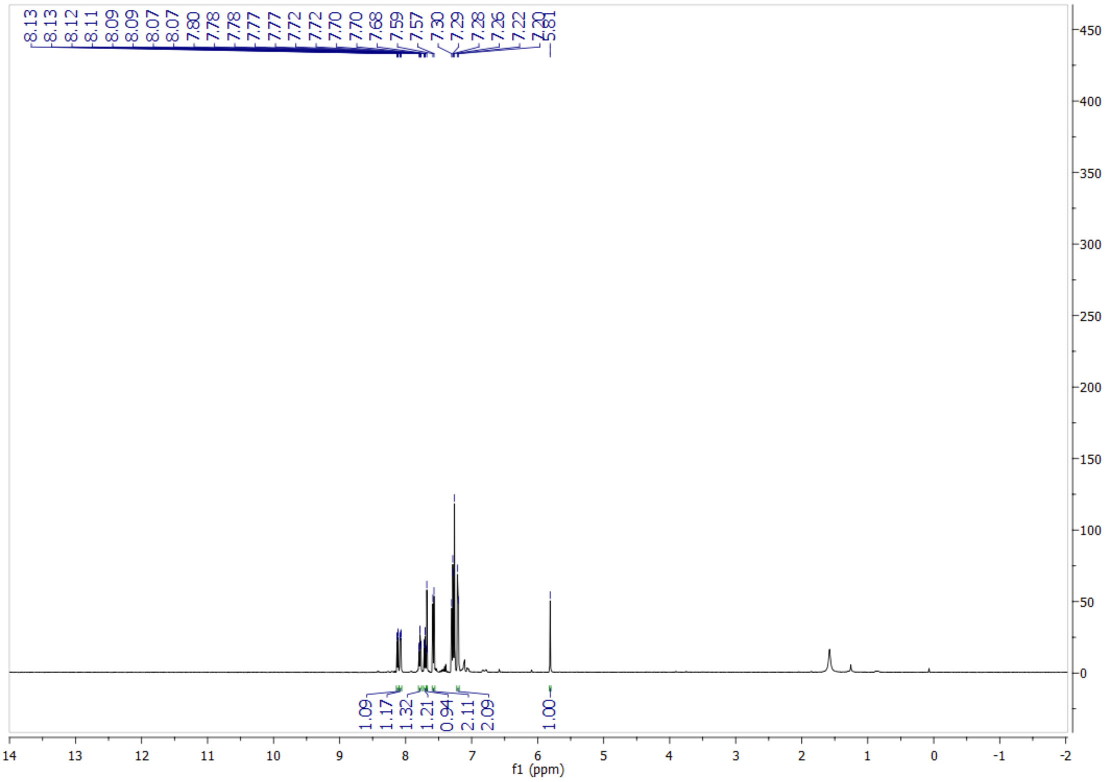

^{1}H NMR for **1a**

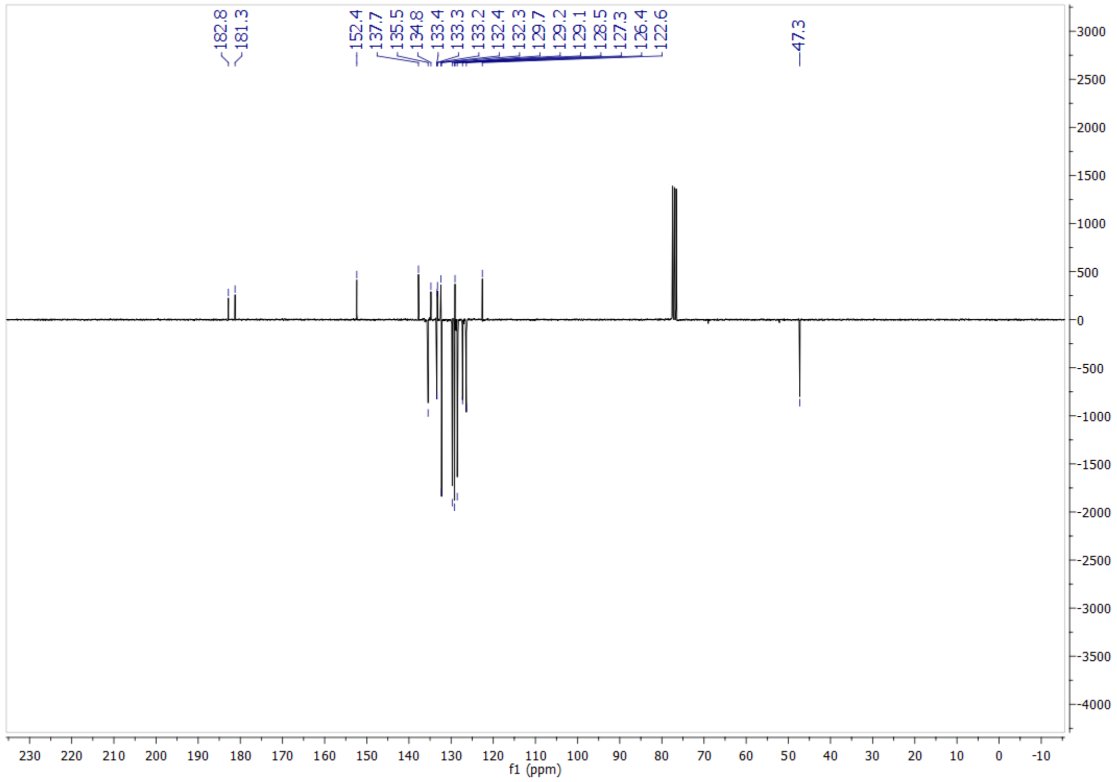

^{13}C NMR/APT for **1a**

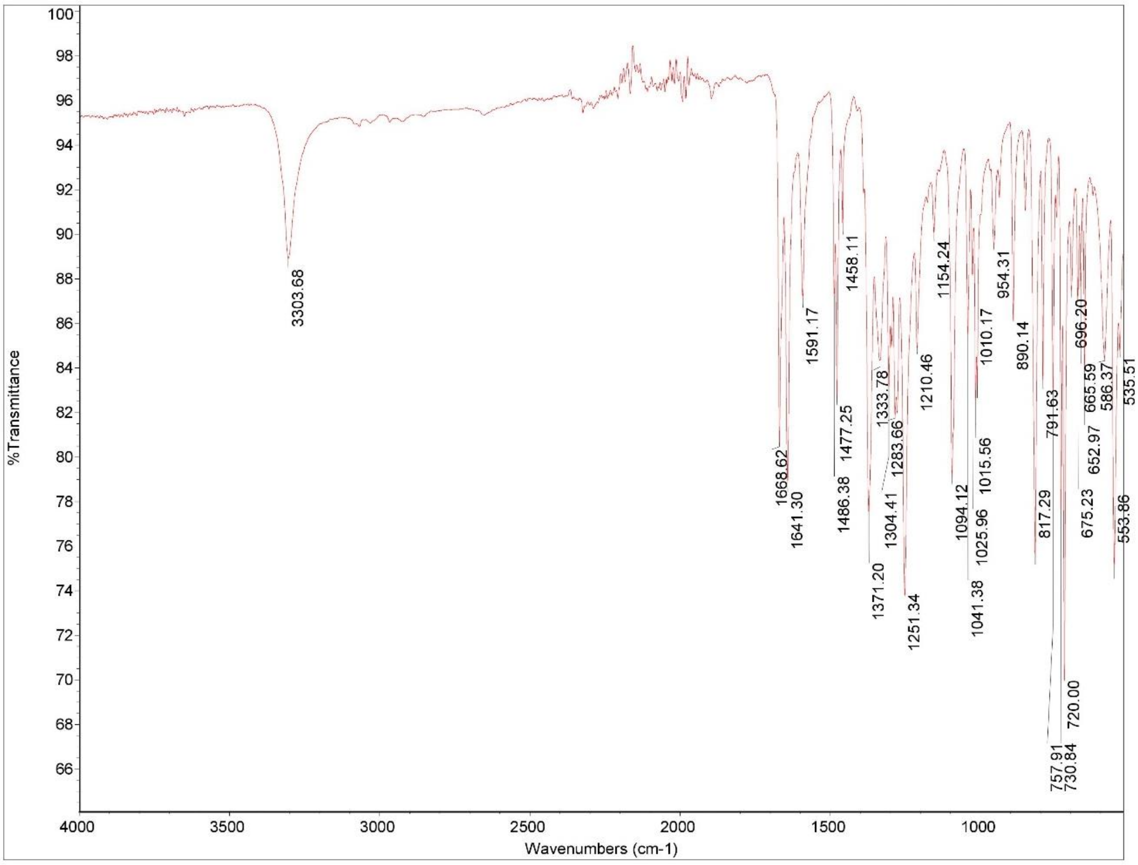

IR for **1a**

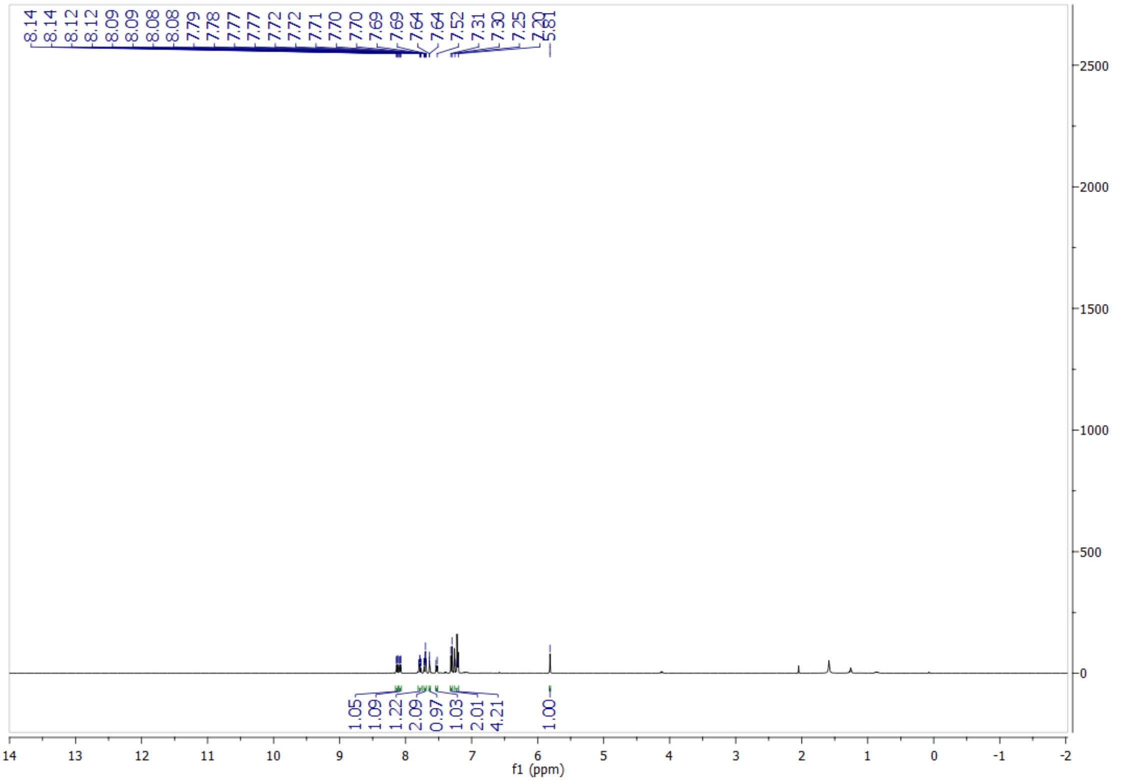

^{1}H NMR for **1b**

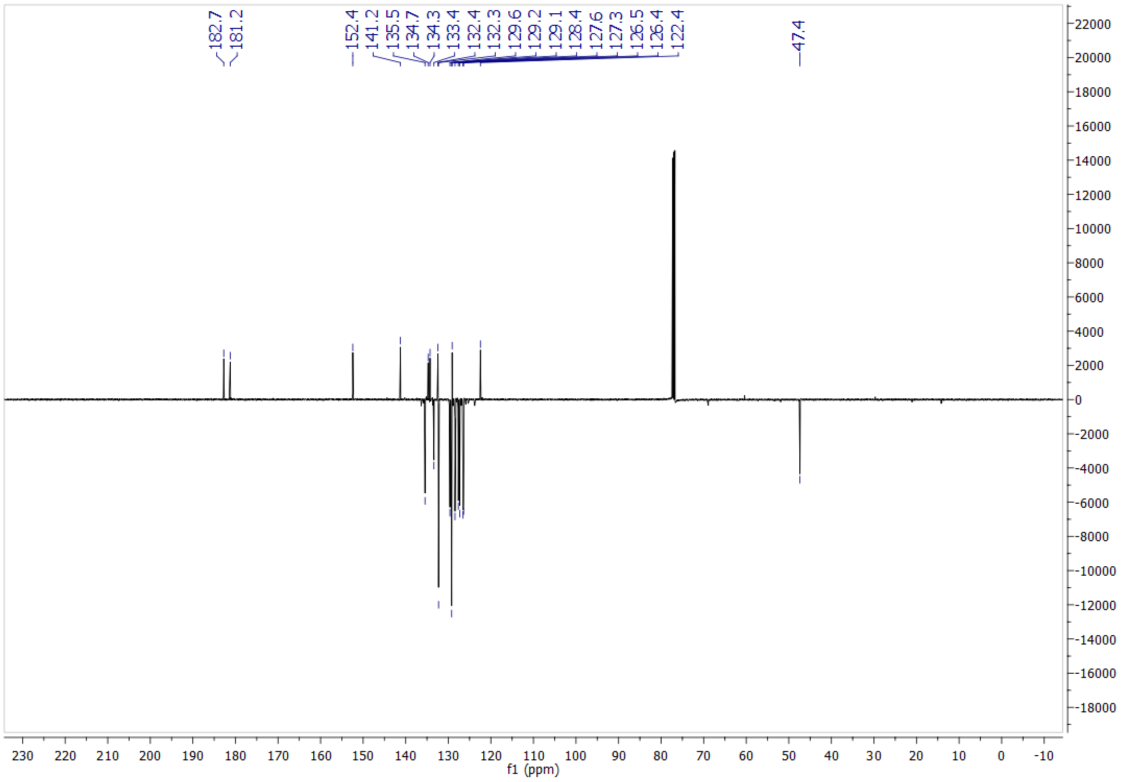

^{13}C NMR/APT for **1b**

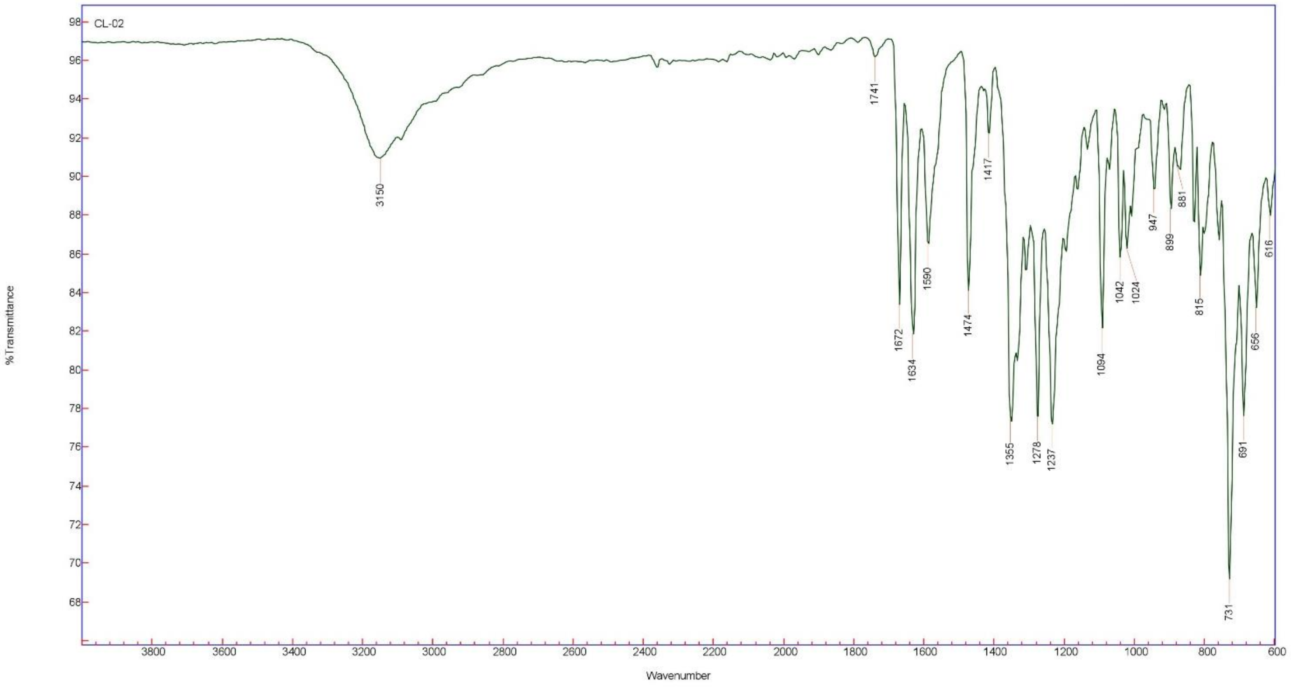

IR for **1b**

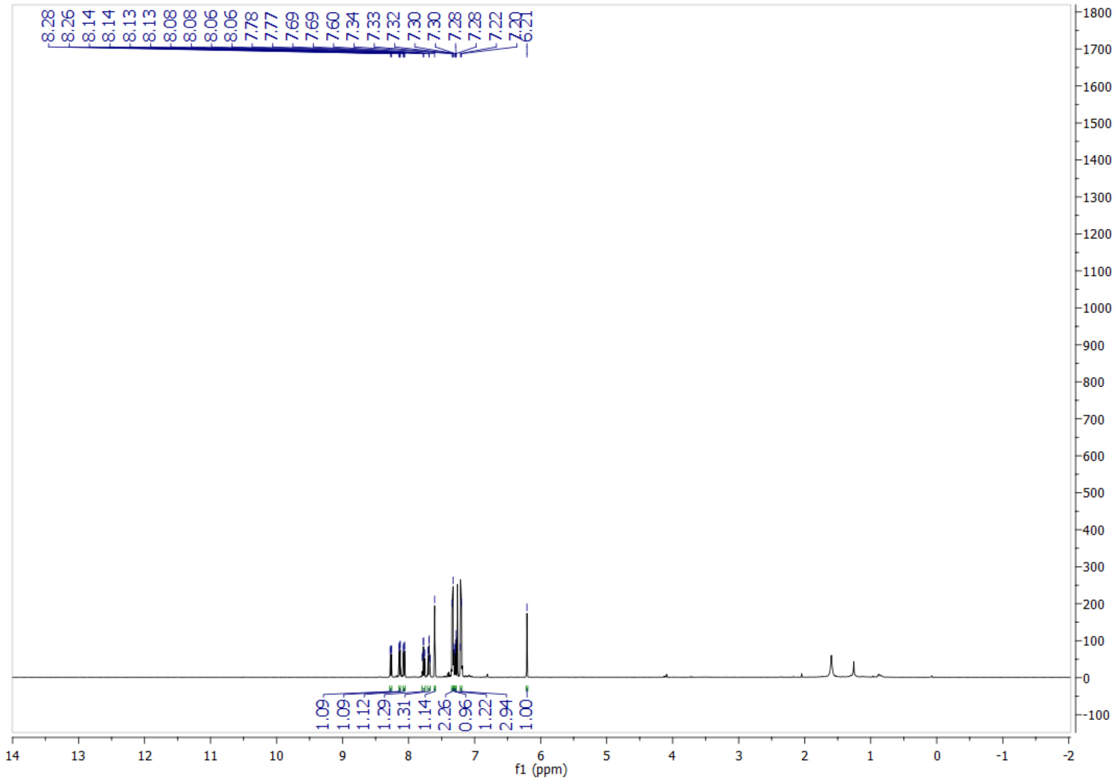

^{1}H NMR for **1c**

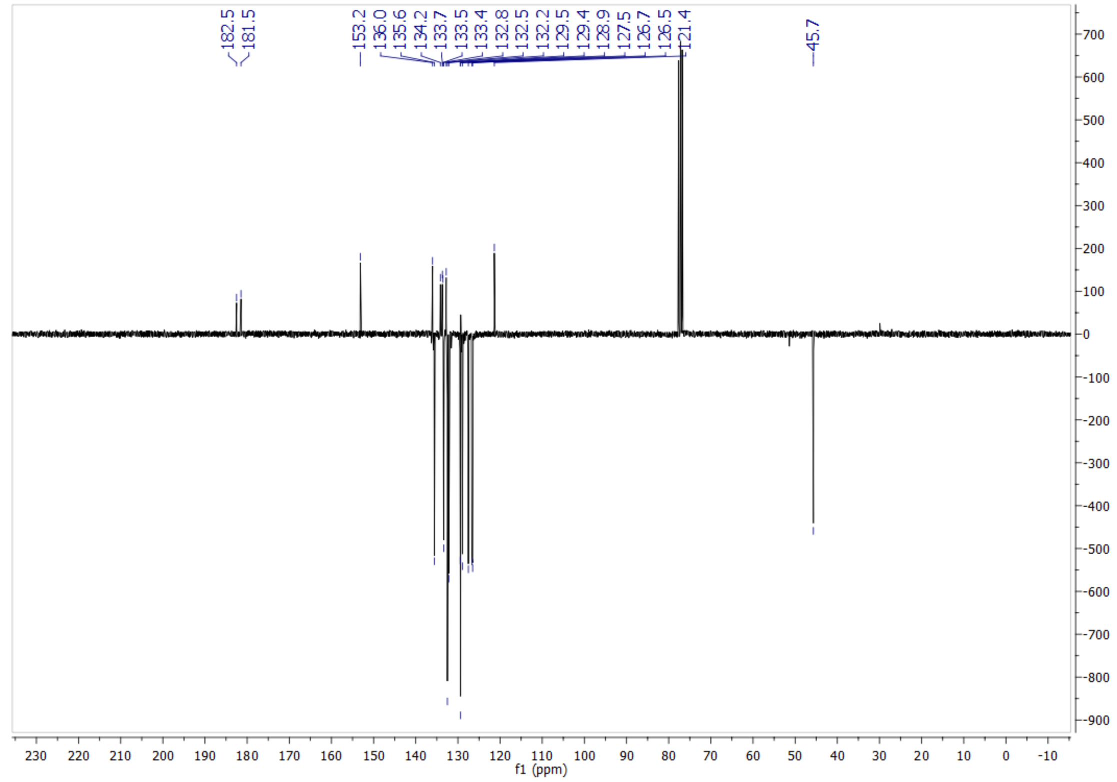

^{13}C NMR/APT for **1c**

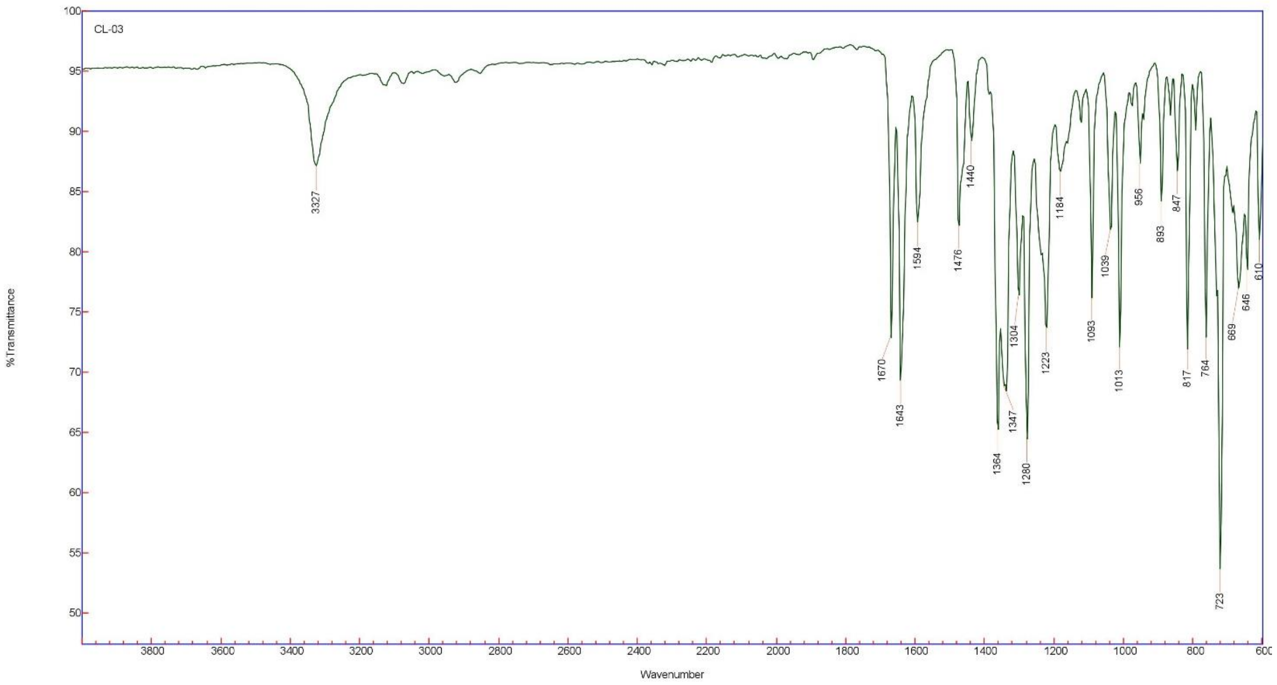

IR for **1c**

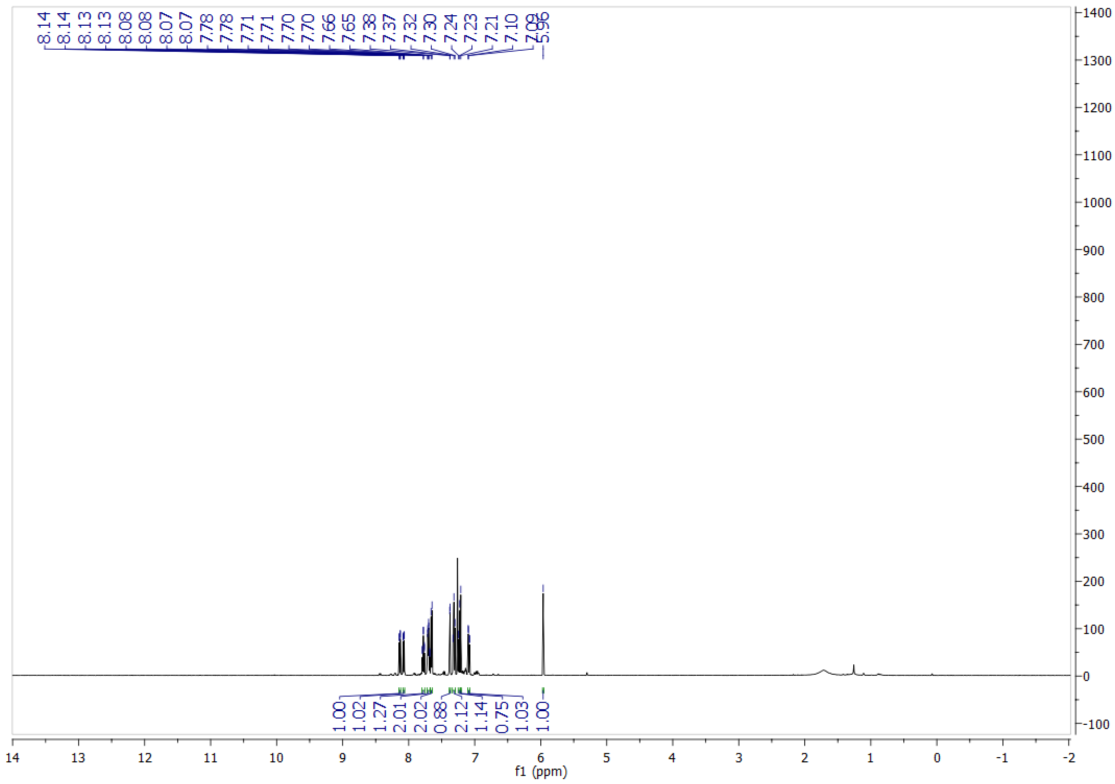

^{1}H NMR for **1d**

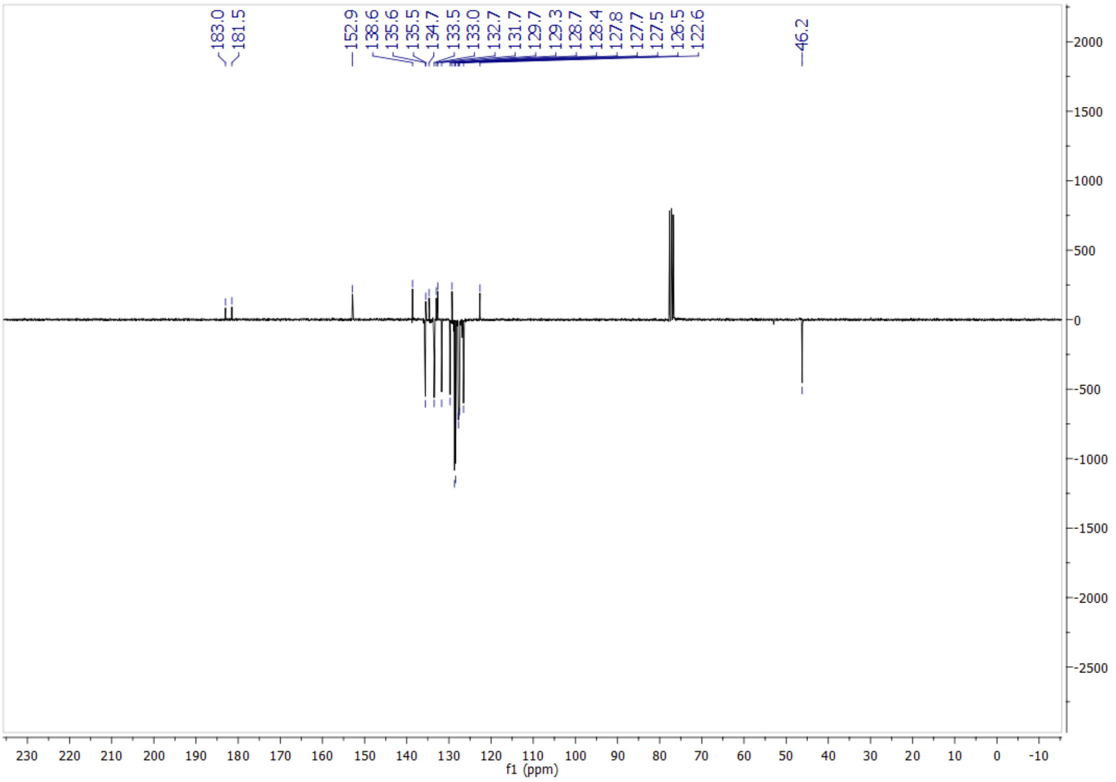

^{13}C NMR/APT for **1d**

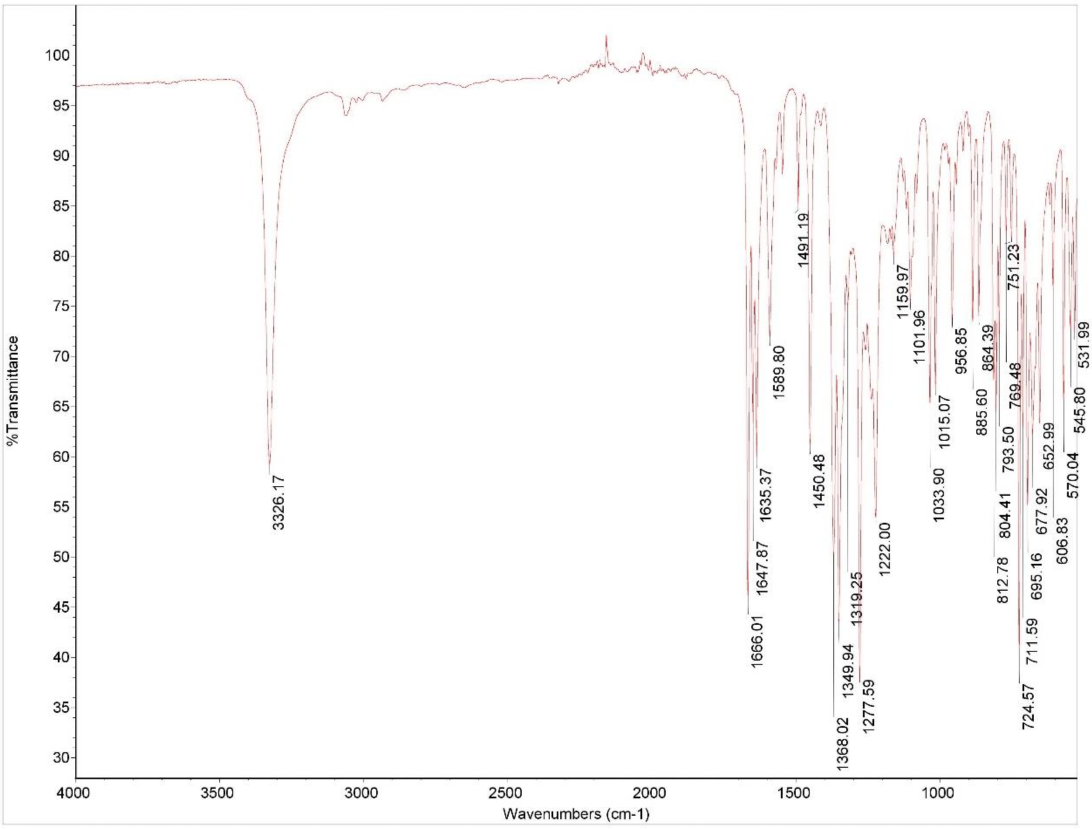

IR for **1d**

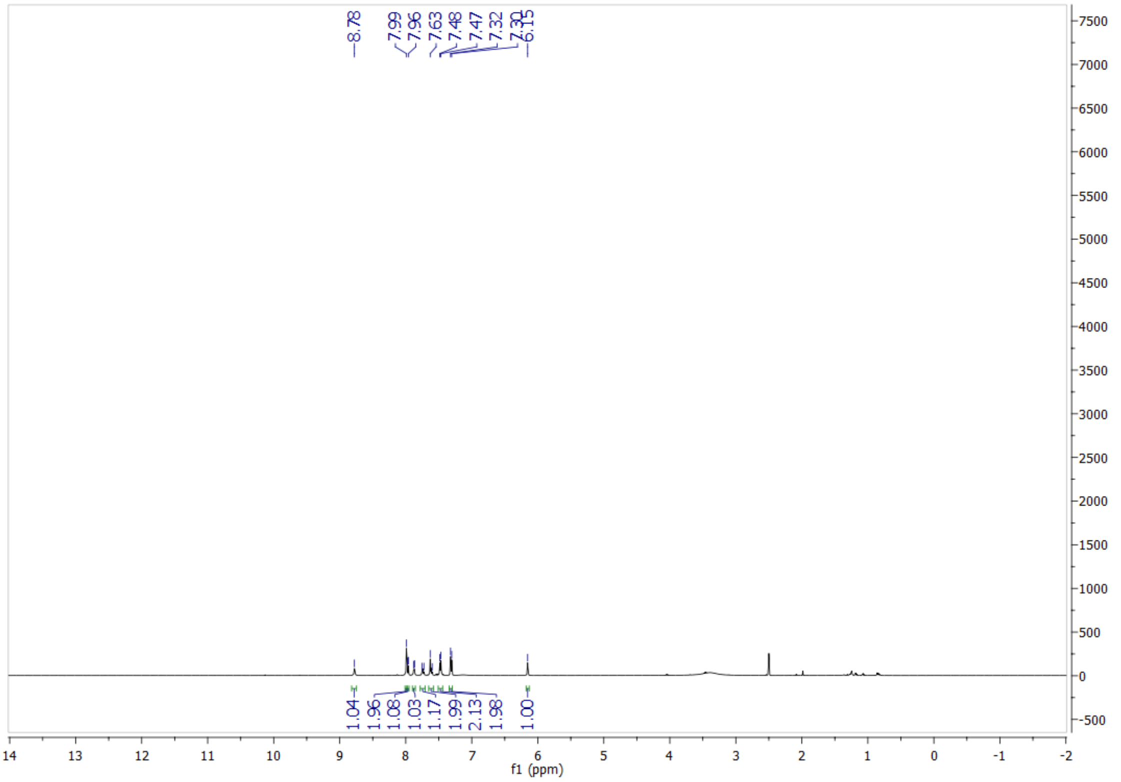

^{1}H NMR for **1e**

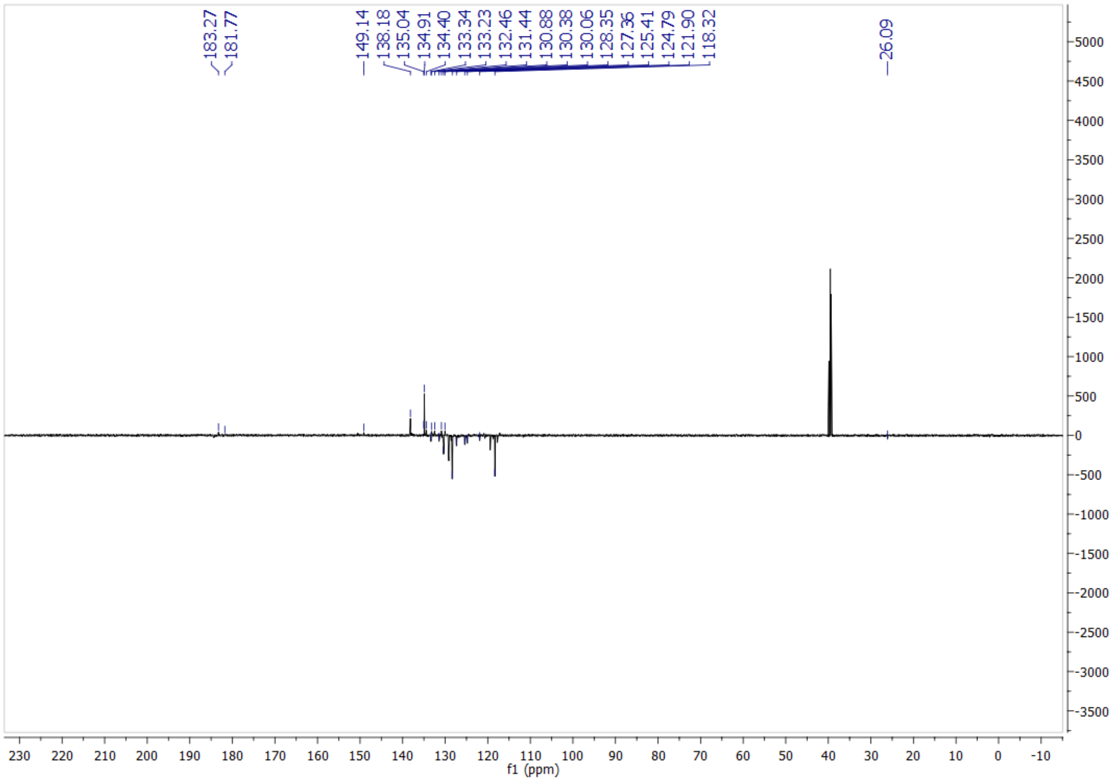

^{13}C NMR/APT for **1e**

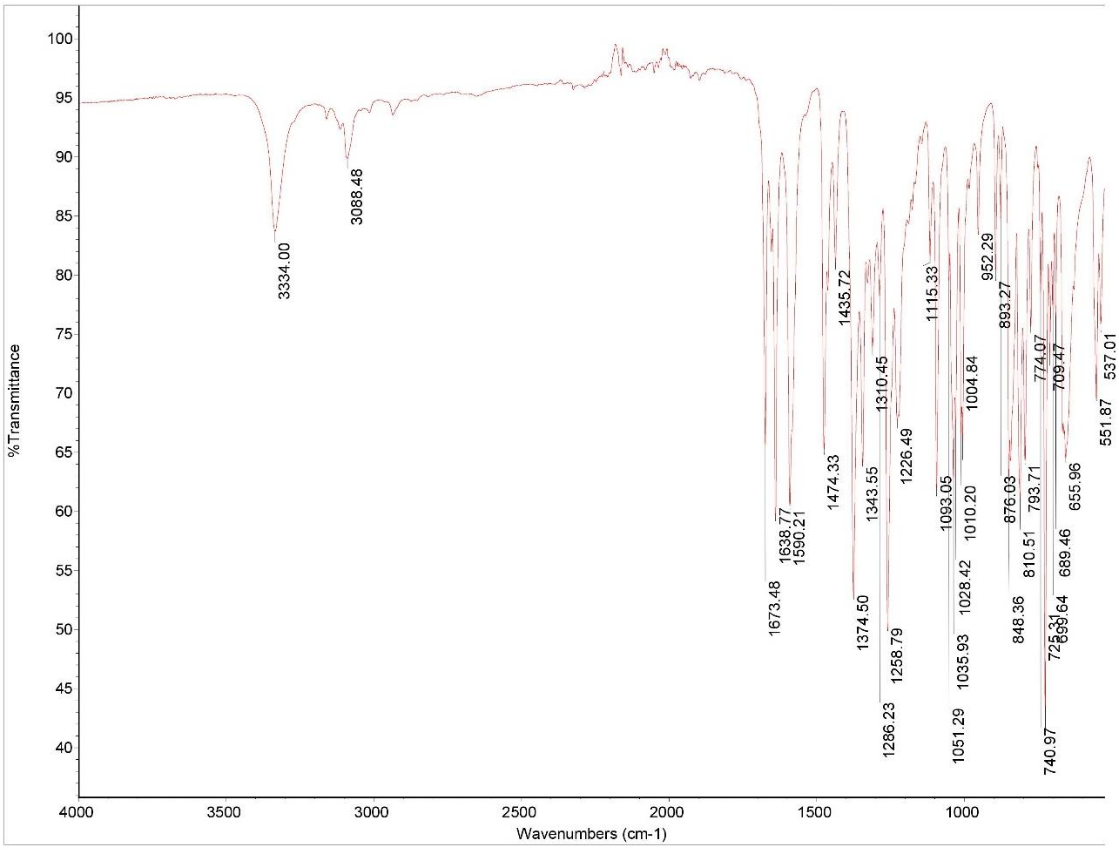

IR for **1e**

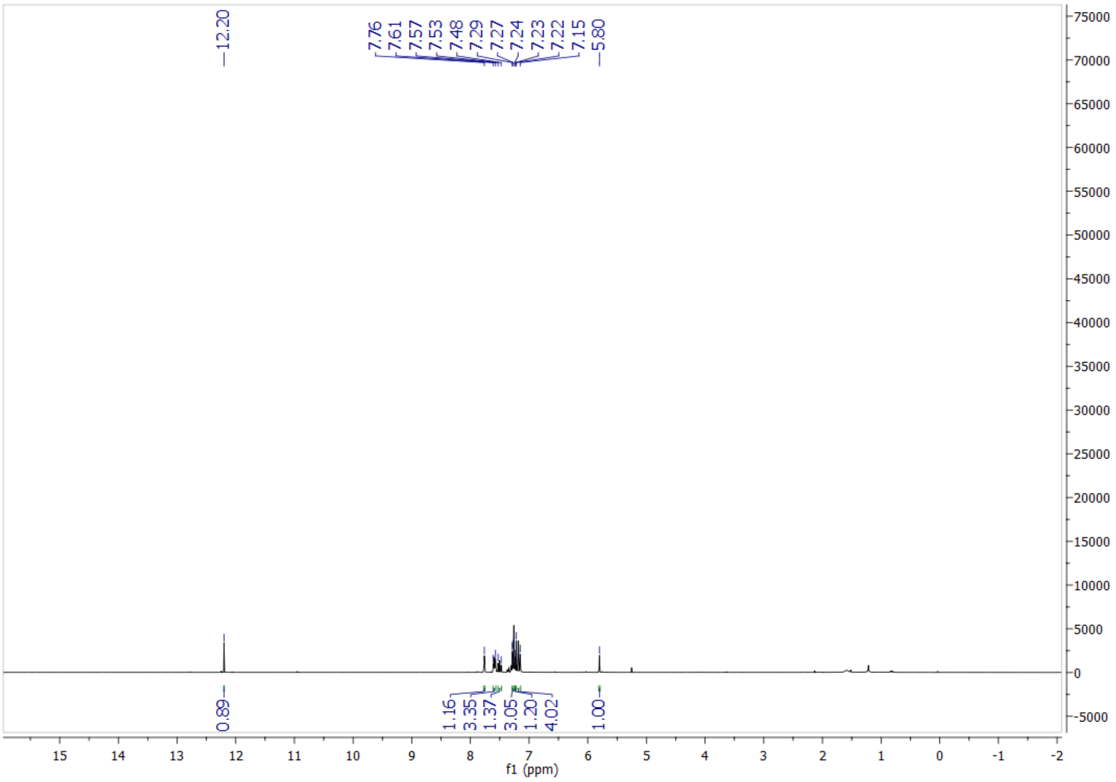

^{1}H NMR for **1f**

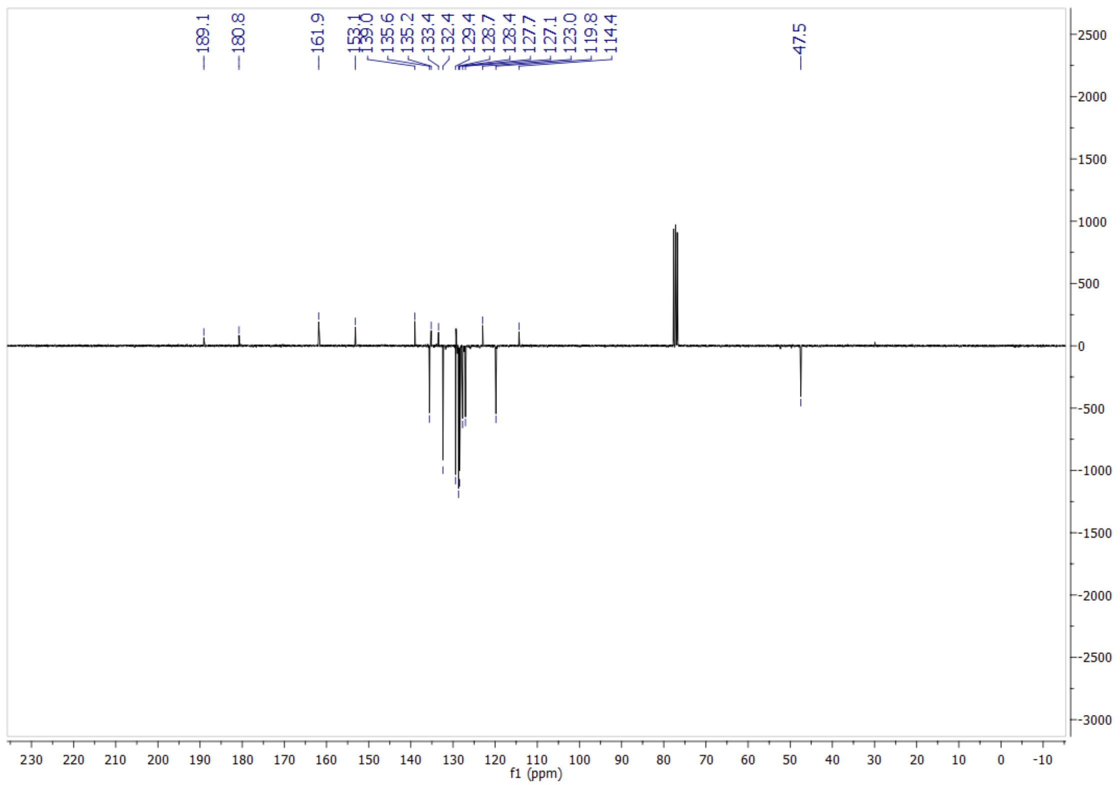

^{13}C NMR/APT for **1f**

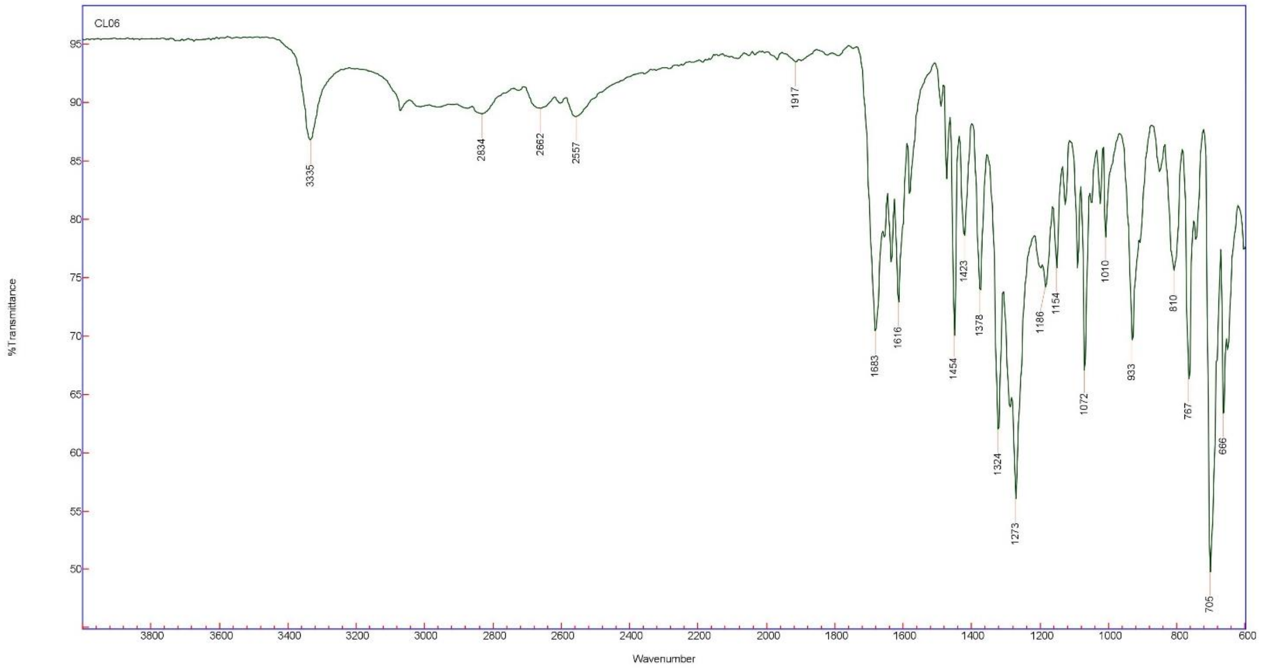

IR for **1f**

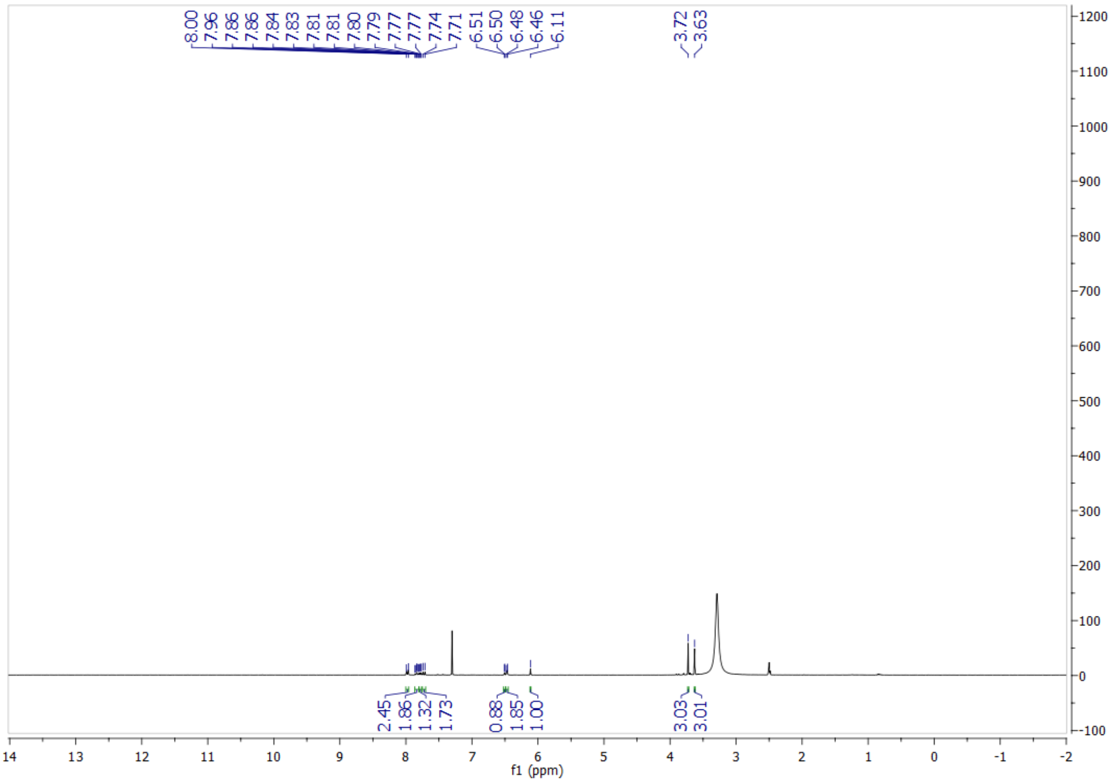

^{1}H NMR for **1h**

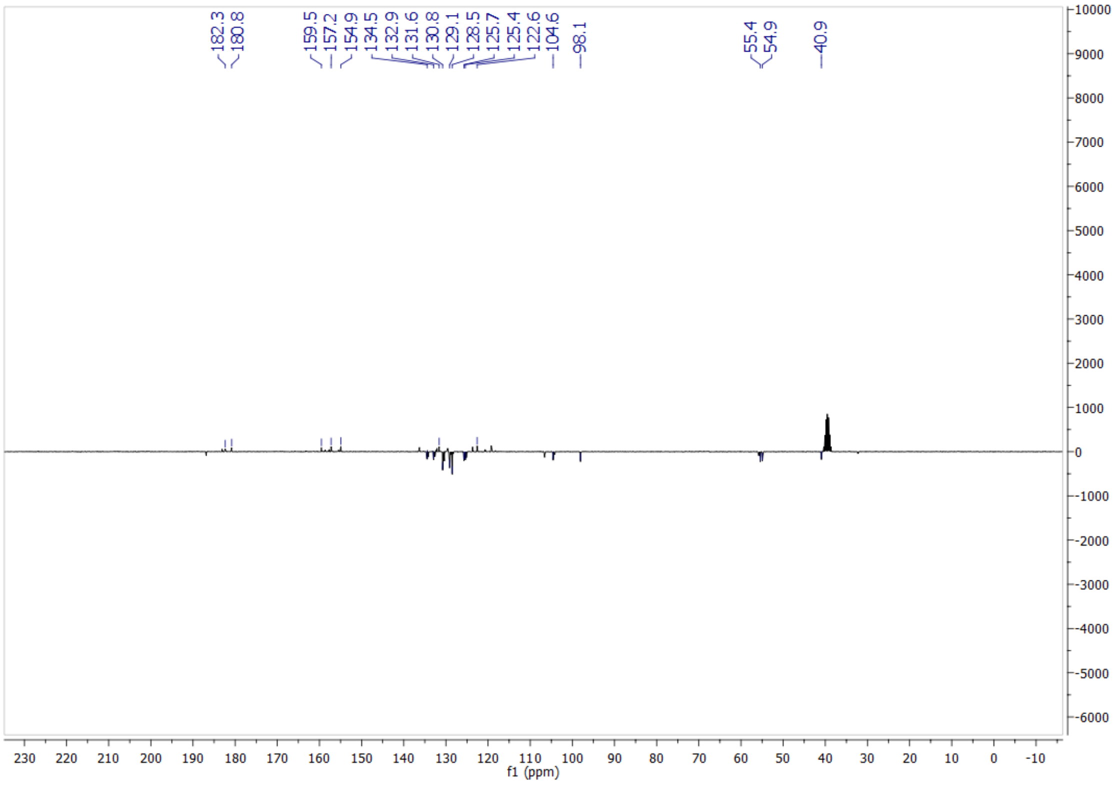

^{13}C NMR/APT for **1h**

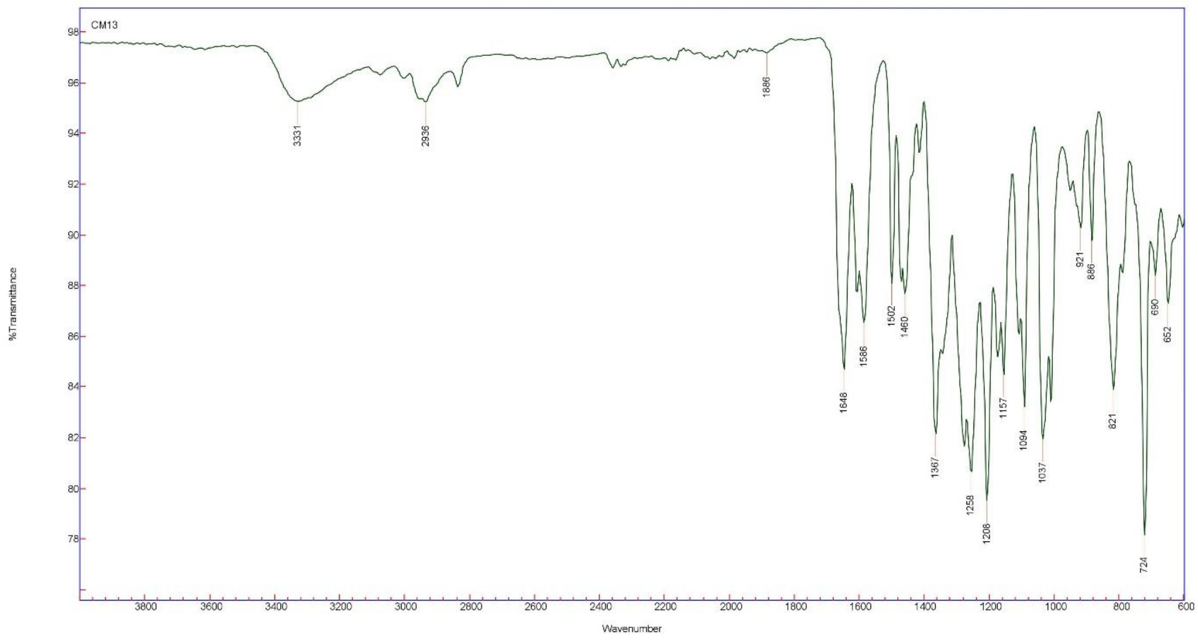

IR for **1h**

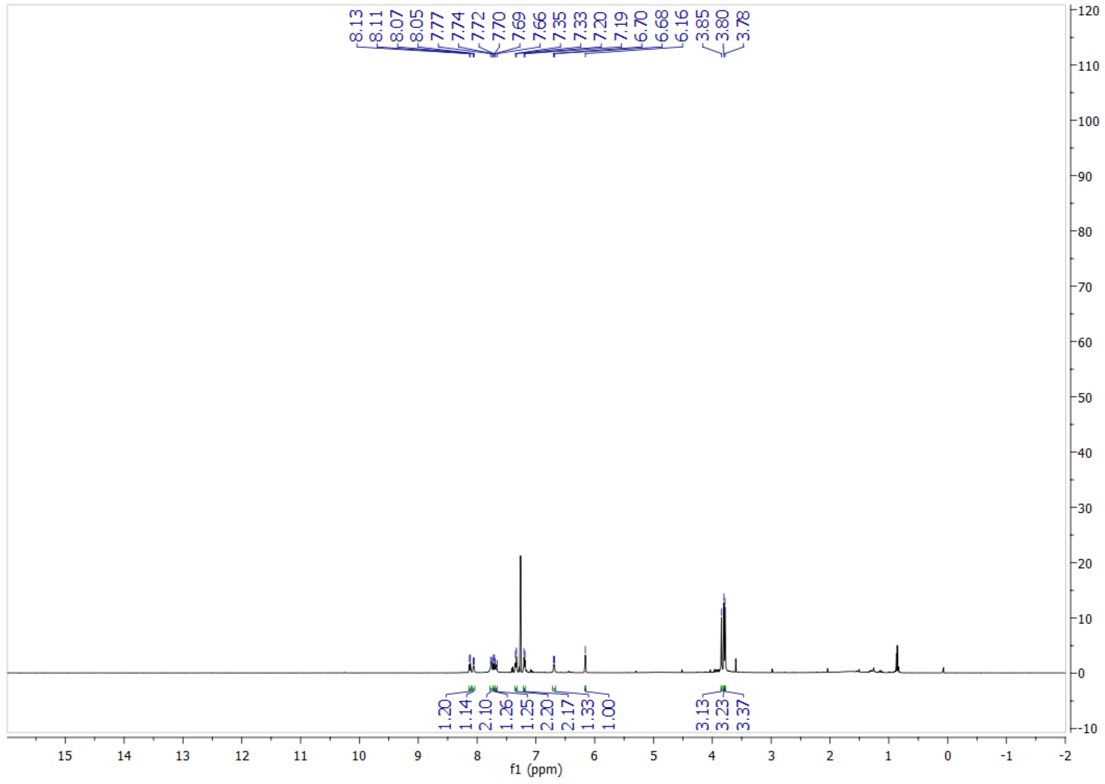

^{1}H NMR for **1i**

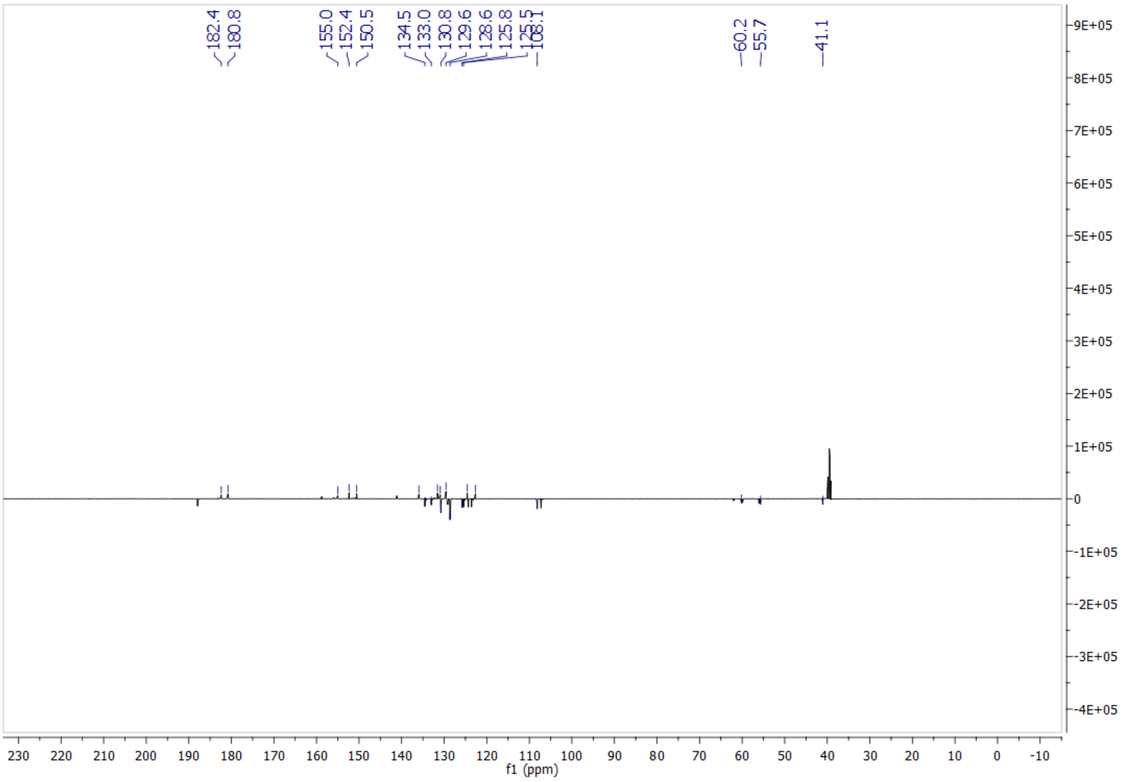

^{13}C NMR/APT for **1i**

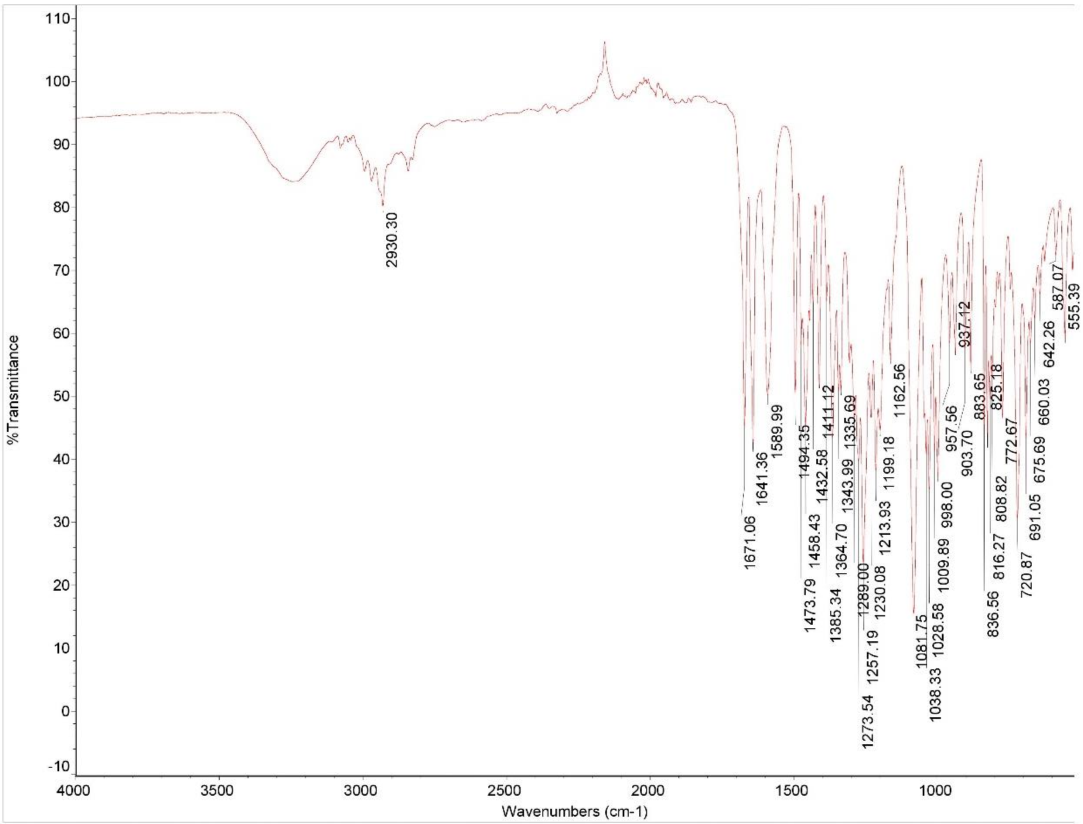

IR for **1i**
